# Whole-body integration of gene expression and single-cell morphology

**DOI:** 10.1016/j.cell.2021.07.017

**Published:** 2021-09-02

**Authors:** Hernando M. Vergara, Constantin Pape, Kimberly I. Meechan, Valentyna Zinchenko, Christel Genoud, Adrian A. Wanner, Kevin Nzumbi Mutemi, Benjamin Titze, Rachel M. Templin, Paola Y. Bertucci, Oleg Simakov, Wiebke Dürichen, Pedro Machado, Emily L. Savage, Lothar Schermelleh, Yannick Schwab, Rainer W. Friedrich, Anna Kreshuk, Christian Tischer, Detlev Arendt

**Affiliations:** 1 Developmental Biology Unit, European Molecular Biology Laboratory (EMBL), Meyerhofstrasse 1, Heidelberg 69117, Germany; 2 Sainsbury Wellcome Centre for Neural Circuits and Behaviour, Howland Street 25, London W1T 4JG, UK; 3 Cell Biology and Biophysics Unit, European Molecular Biology Laboratory (EMBL), Meyerhofstrasse 1, Heidelberg 69117, Germany; 4 Heidelberg Collaboratory for Image Processing, Institut für Wissenschaftliches Rechnen, Ruprecht Karls Universität Heidelberg, Heidelberg, Germany; 5 Friedrich Miescher Institute for Biomedical Research, Maulbeerstrasse 66, Basel 4058, Switzerland; 6 Princeton Neuroscience Institute, Princeton University, Washington Road, Princeton, NJ 08544, USA; 7 Department of Neuroscience and Developmental Biology, University of Vienna, Althanstr. 14, Vienna 1090, Austria; 8 Centre for Ultrastructural Imaging, King’s College London, New Hunt’s House Guy’s Campus, London SE1 1UL, UK; 9 Micron Advanced Bioimaging Unit, Department of Biochemistry, University of Oxford, South Parks Road, Oxford OX1 3QU, UK; 10 Cell Biology and Biophysics Unit/Electron Microscopy Core Facility, European Molecular Biology Laboratory (EMBL), Meyerhofstrasse 1, Heidelberg 69117, Germany; 11 Centre for Bioimage Analysis, Core Facilities, European Molecular Biology Laboratory (EMBL), Meyerhofstrasse 1, Heidelberg 69117, Germany; 12 Collaboration for joint PhD degree between EMBL and Heidelberg University, Faculty of Biosciences; 13 Electron Microscopy Facility, Faculty of Biology and Medicine, University of Lausanne, biophore, quartier Sorges, 1015 Lausanne, Switzerland; 14 Paul Scherrer Institute (PSI), Forschungsstrasse 111, 5232 Villigen PSI, Switzerland; 15 Centre for Organismal Studies (COS), University of Heidelberg, Heidelberg, Germany

**Keywords:** volume electron microscopy, image registration, automatic segmentation, gene expression atlas, *Platynereis dumerilii*, cell types, multimodal data integration, machine learning, mushroom bodies, telencephalon

## Abstract

Animal bodies are composed of cell types with unique expression programs that implement their distinct locations, shapes, structures, and functions. Based on these properties, cell types assemble into specific tissues and organs. To systematically explore the link between cell-type-specific gene expression and morphology, we registered an expression atlas to a whole-body electron microscopy volume of the nereid *Platynereis dumerilii*. Automated segmentation of cells and nuclei identifies major cell classes and establishes a link between gene activation, chromatin topography, and nuclear size. Clustering of segmented cells according to gene expression reveals spatially coherent tissues. In the brain, genetically defined groups of neurons match ganglionic nuclei with coherent projections. Besides interneurons, we uncover sensory-neurosecretory cells in the nereid mushroom bodies, which thus qualify as sensory organs. They furthermore resemble the vertebrate telencephalon by molecular anatomy. We provide an integrated browser as a Fiji plugin for remote exploration of all available multimodal datasets.

## Highlights

• A cellular atlas integrates gene expression and ultrastructure for an entire annelid
• Morphometry of all segmented cells, nuclei, and chromatin categorizes cell classes
• Molecular anatomy and projectome of head ganglionic nuclei and mushroom bodies
• An open-source browser for multimodal big image data exploration and analysis

## Graphical abstract

**Figure:**
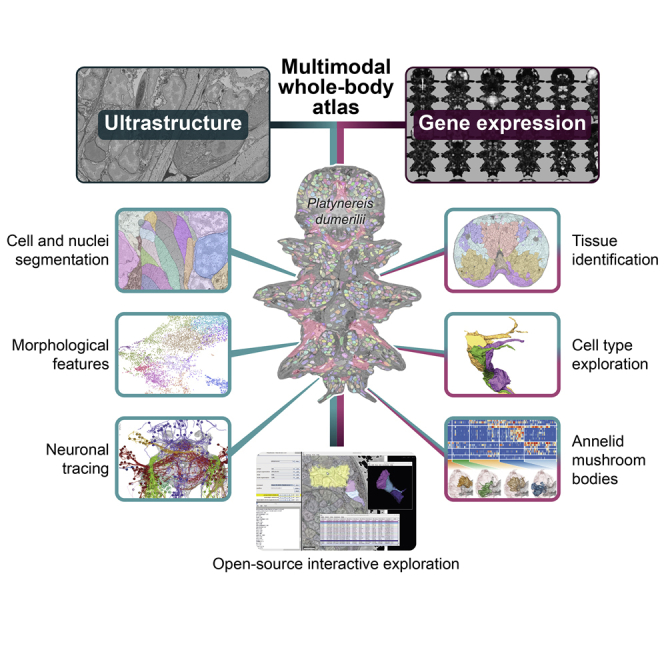

## Teaser

A framework for integrating cellular-resolution gene expression and cell morphological information at full-organism scale is provided for the marine annelid Platynereis dumerilii

## Introduction

Cells are the basic units of life. In multicellular organisms, distinct genes are expressed in different cells, producing individual traits that define cell types (Arendt et al., 2016). Deciphering how genotype is decoded into cellular phenotype is thus critical to understand the structure and function of an entire body. Toward this goal, we need to establish the link between expression profiles and cellular morphologies. This requires techniques that permit the integration of genetic and phenotypic information for all cells of the body. On one hand, volume electron microscopy (EM) produces 3D ultrastructural data for cells and tissues with unprecedented coherency and detail (see Titze and Genoud, 2016 for review). On the other, spatial single-cell omics techniques have revolutionized expression profiling (Lein et al., 2017).

In established model species, the use of transgenic lines permits the combined molecular, morphological, and functional interrogation of cell types and enables multimodal databases and atlases (Clements et al., 2020; Milyaev et al., 2012; Sunkin et al., 2013; Altun et al., 2002). This includes the use of genetic tools to link connectomics and transcriptomics for the full central nervous system of the fly (Bates et al., 2019). This strategy is, however, very costly and hard to scale to the full organism. More recently, single-cell sequencing has enabled cell-type classification for any organism but at the cost of losing anatomical information. The objective of this study is to provide a framework for generating atlases that integrate transcriptional and morphological information for all cells in an entire animal body, using techniques that are not species specific. To this end, we introduce a pipeline integrating gene expression and cellular ultrastructure for the 6-days post-fertilization (dpf) young worm of the marine annelid *Platynereis dumerilii*. At this stage, *Platynereis* already exhibits a rich and differentiated set of cell types, which is comparable to that of many bilaterians, including vertebrates. However, because each cell type comprises a few cells only, the overall number of cells remains small. This goes in concert with a considerable stereotypy of *Platynereis* development and differentiation: the developmental lineage is invariant (Vopalensky et al., 2019), and differentiated larvae and young worms resemble each other down to the cellular detail (Asadulina et al., 2012; Randel et al., 2015; Tomer et al., 2010; Vergara et al., 2017).

We first acquired a serial block-face scanning EM (SBEM) volume and introduced a semi-automated multi-scale approach for cellular segmentation of the whole animal. This allowed us to quantitatively characterize the morphology of all body cells. This revealed classes of cells, such as neurons, muscle, or epithelial cells, that not only differ in shape or cytoplasmic features but also in chromatin characteristics. We then expanded an existing gene expression atlas for the 6-dpf stage (Vergara et al., 2017) and registered it to the segmented EM volume. For the first time, this enabled us to assign gene expression information to the segmented cells of an entire body and to cluster these according to expression profile. We demonstrate that such genetically defined clusters respect morphological boundaries and correspond to distinct ganglionic nuclei with coherent axonal projections. Our multimodal data reveal a fundamental duality in the *Platynereis* brain of segmentally iterated parts, such as sensory appendages, on one hand, and unique parts, such as the sensory-neurosecretory dorsal brain, on the other. Focusing on the mushroom bodies (MBs)—the invertebrate associative centers—we detect a proliferative center generating neurons that molecularly resemble vertebrate telencephalic interneurons. We provide the open-source platform “PlatyBrowser” to integrate, explore, and analyze multimodal data at the level of cell types, tissues, and organs. We expect the tools presented here to be transferable to EM volumes of other animals with some degree of developmental stereotypy, enabling the comparison of multimodal cell type catalogs across organisms.

## Results

### A whole-body SBEM dataset

**Figure 1:**
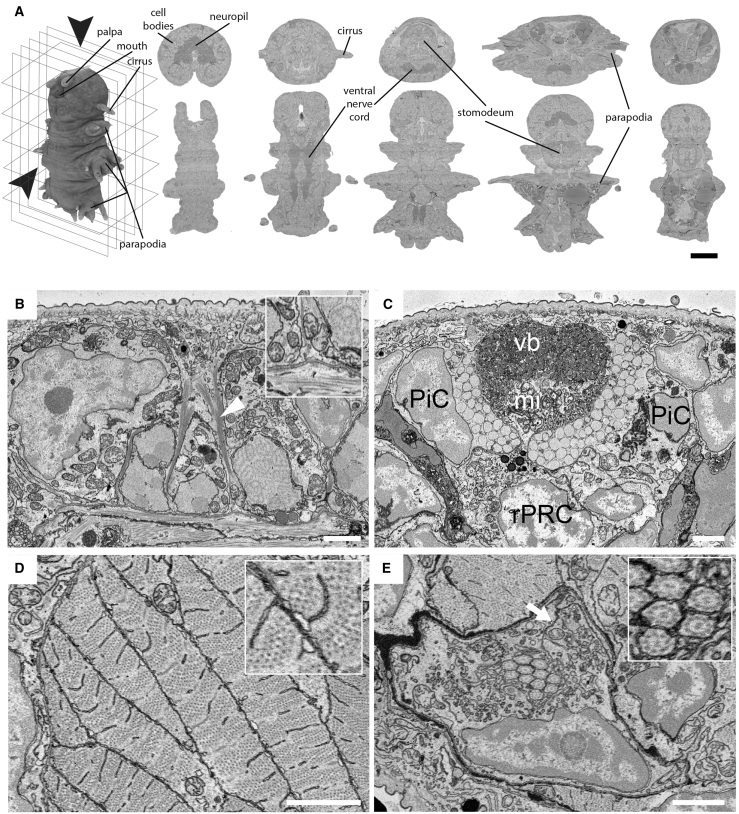
A whole-body serial block-face scanning electron microscopy dataset (A) The SBEM dataset can be observed in all orientations (e.g., transversal plane in top row or the horizontal plane in bottom row; scale bar: 50 μm). (B–E) Fine ultrastructure at native resolution (10 nm pixel-size x/y; scale bars: 2 μm). (B) Epithelial cell, interfacing cuticle, and underlying muscle. Bundles of cytoskeletal filaments (arrowhead) form part of the attachment complex (inset). (C) The adult eye forms a pigment cup composed of pigment cells (PiCs) and rhabdomeric photoreceptors (rPRCs), which extend a distal segment of microvillar projections (mi) for light detection. In the center of the pigment cup is the vitreous body (vb). (D) Longitudinal muscle fibers are cut transversally, displaying cross-sections of the sarcomere as well as of the sarcoplasmic reticulum that contacts the plasma membrane (inset). (E) Cross-section of the distal part of the nephridia, highlighting the autocell junction (arrow). The lumen houses a bundle of motile cilia (with 9+2 microtubules, inset) contributed by each nephridial cell. (B)–(E) are snapshots that can be retrieved via the PlatyBrowser “Bookmark” function. See also Figure S1.

**Figure S1:**
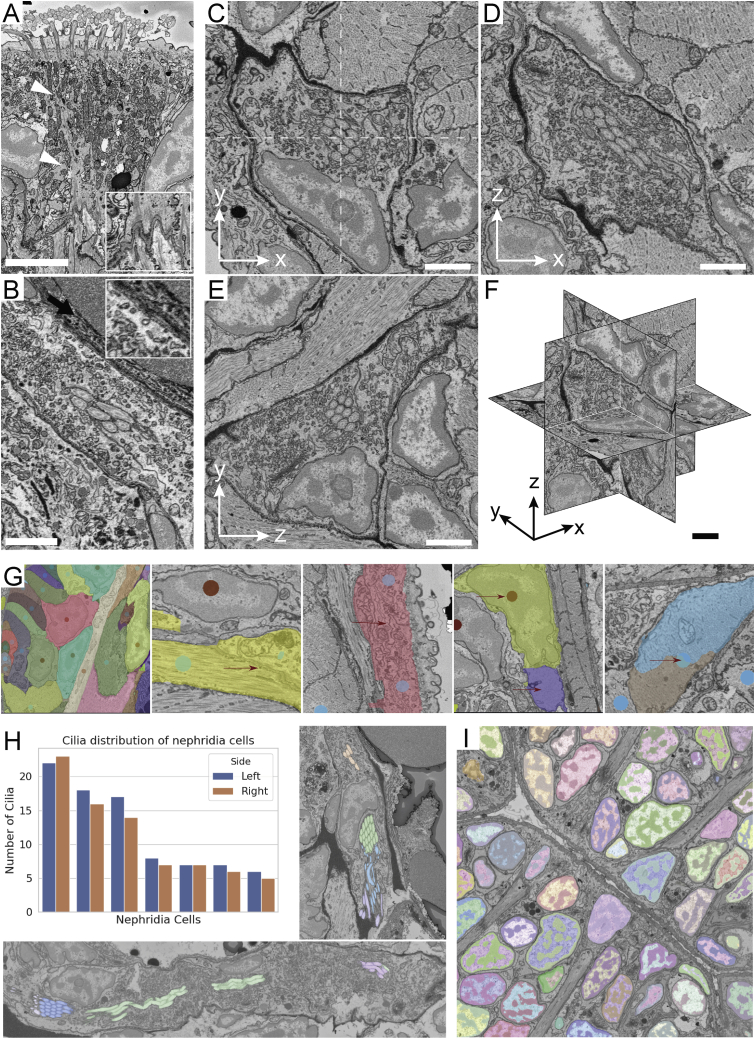
Ultrastructure of different cell types, segmentation validation, and ultrastructure segmentation, related to Figures 1 and 2 A. Ciliated support cells which are part of the nuchal organ in *Platynereis*. Cytoskeletal fibers (arrowheads) and anchoring points at the junction of the support cell and the underlying muscles are visible (inset) (Scale bar 5 μm). B. Cell of the nephridia which contains a lumen occupied by motile cilia. These cells contain numerous vesicles responsible for various forms of cell transport. A site of endocytosis, identified by the presence of a clathrin-coated pit is highlighted (arrow)(inset) (Scale bar 2 μm). C-F. Orthogonal projections of the image displayed in Figure 1E - scaling factor of 2.5x was applied to the Z plane to get an isotropic render (Scale bars 2 μm). G: Nuclei and cells were annotated by domain experts for 8 slices (4 transversal, 4 horizontal), see leftmost image for example annotations. We used these annotations to find false merge errors, see the two middle images with arrows highlighting the cell membrane not picked up, and false split errors, see two rightmost images with arrows highlighting parts of the cell that were split off, in the automated segmentation. H: The distribution of cilia per cell for the nephridia on both sides is stereotypical as can be seen from the plot. Cilia in a given cross-section of the lumen almost exclusively start off from the same cell, see segmented cilia colored by their cell of origin overlaid on the EM; upper image shows a cross section of the right nephridium, lower image of the left nephridium. I: Chromatin segmentation overlaid on the EM. The dark phase (classical heterochromatin + nucleolus) and the light phase (classical euchromatin) are segmented. Panels A, B and I are available as bookmarks in the PlatyBrowser.

An EM image stack of a complete 6-dpf young *Platynereis* worm was collected by SBEM at a pixel size (x/y) of 10 nm and 25 nm section thickness (z), resulting in 11,416 planar images made of >200,000 tiles for a total size of 2.5 TB. This dataset enabled detailed analyses of overall anatomy and ultrastructural detail throughout the body (Figures 1 and S1). For example, attachment complexes are identified in epithelial (Figure 1B) and ciliated support cells (Figure S1A). Made of intermediate filaments, these complexes connect to the underlying muscle layer and withstand the mechanical forces exerted at this interface. In the rhabdomeric photoreceptors of the eye, characteristic microvilli and sub-microvillar cisternae can be detected (Figure 1C). Individual myosin filaments are visible in *Platynereis* muscles, which lack the t-tubule system present in vertebrates and instead have an extended sarcoplasmic reticulum (Rosenbluth, 1968; Figure 1D).

**Figure 2:**
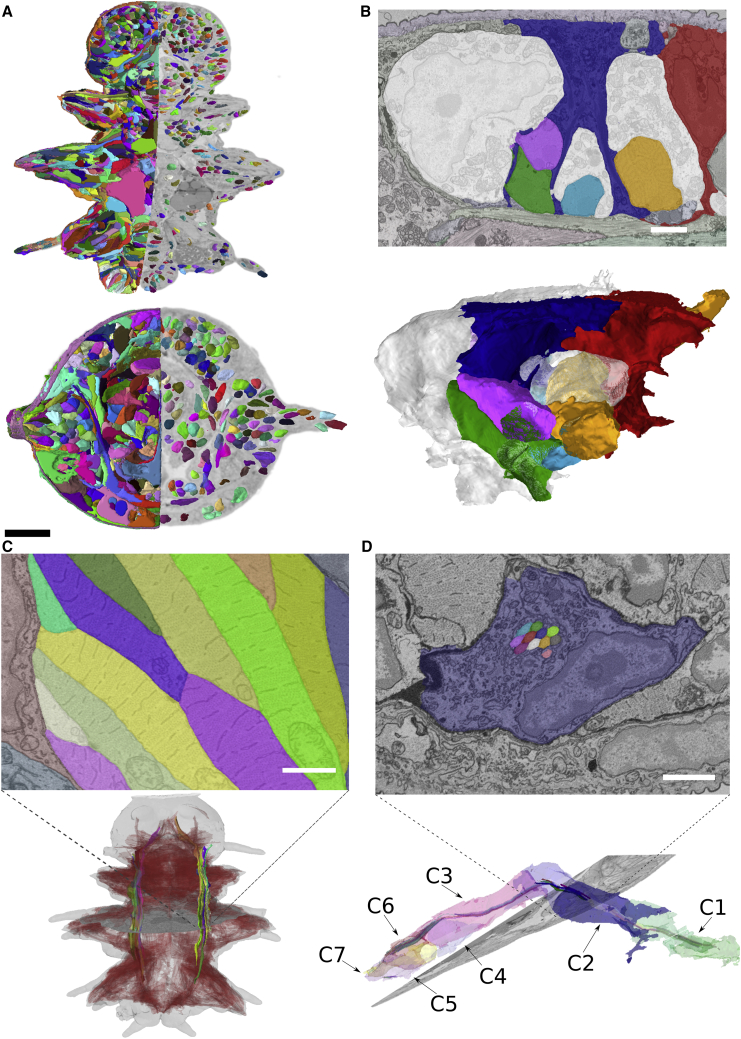
Segmentation of nuclei and cells (A) Horizontal and transverse sections with 3D renderings of cells (left) and nuclei (right). (B) Intertwined epithelial cells shown as EM-overlaid colored segments and 3D renderings. (C) Segmentation of longitudinal muscles highlighted in the bottom rendering, with other muscles rendered in the background. (D) Cross-section of segmented nephridial cell and individually colored cilia (top). (Bottom) 3D rendering of one nephridium with each cilium colored as the cell it belongs to. Bookmarks for views in (B), (C), and (D) (corresponding to Figures 1B, 1D, and 1E) are available in the PlatyBrowser. The scale bar in (A) corresponds to 50 μm and in (B)–(D) to 2 μm. See also Figure S1.

The paired larval nephridia (Hasse et al., 2010) are found adjacent to the longitudinal muscles between the 2nd and 3rd segment (Figures 1E, 2D, and S1B). Each composed of 7 cells, they connect the coelomic cavity to the outside. All cells form a lumen, which is closed off via auto-cell junctions (Figure 1E) and into which they protrude motile cilia (with 9 + 2 microtubules; Figure 1E, inset). The terminal cell of each nephridium forms the nephridiopore, which is surrounded by microvilli. We also identify multiple transport vesicles (Bartolomaeus and Quast, 2005) and sites of endocytosis from the coelomic cavity (Figure S1B, inset). Similar ultrastructural analysis can be performed on other cells, illustrating the resolution power for the full 6-dpf *Platynereis*.

### Segmentation of nuclei and cells

We aimed to segment every cell, nucleus, and selected ultrastructure from the EM volume. Nuclei were segmented with the Mutex Watershed algorithm (Wolf et al., 2018). For cell segmentation, we used the Lifted Multicut framework (Pape et al., 2019) that uses boundary predictions from a convolutional neural network, complemented by top-down constraints derived from the nucleus segmentation and additional tissue segmentations provided at lower scale. We corrected 154 merged cells using a semi-automatic approach and proofread the detailed morphology for selected cells with Paintera (Hanslovsky et al., 2020). The segmentation contains 11,402 cells with nuclei (Figure 2A). Segmentations were validated against 8 manually annotated slices (4 transversal and 4 horizontal) distributed throughout the dataset. Here, we found a 99.0% agreement with the automatic nuclear segmentation and a 90.3% agreement with the cellular segmentation (Figure S1G). Figure 2 exemplifies segmentation quality for epidermal cells (Figure 2B), muscles (Figure 2C), and nephridia (Figure 2D). Nuclei sizes ranged from 33.6 to 147.5 μm^3^ and cell sizes from 59.8 to 1,224.6 μm^3^. Note that automated segmentation did not cover neurites.

To assess the quality of cellular segmentation, we focused on the larval nephridia (Figure 2D). Previous transmission EM analysis revealed six cilia constantly present in the nephridial lumen (Hasse et al., 2010) but could not attribute them to individual cells. As proof of principle for comprehensive ultrastructure segmentation, we also segmented the nephridial cilia and showed that each of the 7 cells per side contributes several cilia to the continuous central bundle. The bundle contains 85 and 78 cilia on the left and right side, respectively. We observed a similar distribution of cilia per cell for both sides and found that, for any luminal cross-section, cilia mostly stem from one cell (Figure S1H).

The strong contrast in subnuclear structure allowed the semantic segmentation of chromatin using the nuclear segmentation as a mask (Figure S1I). Nuclei showed a meandering pattern of light and dark subregions, corresponding to classical euchromatin and heterochromatin plus nucleolus.

### Morphological clustering of segmented cells

**Figure S2:**
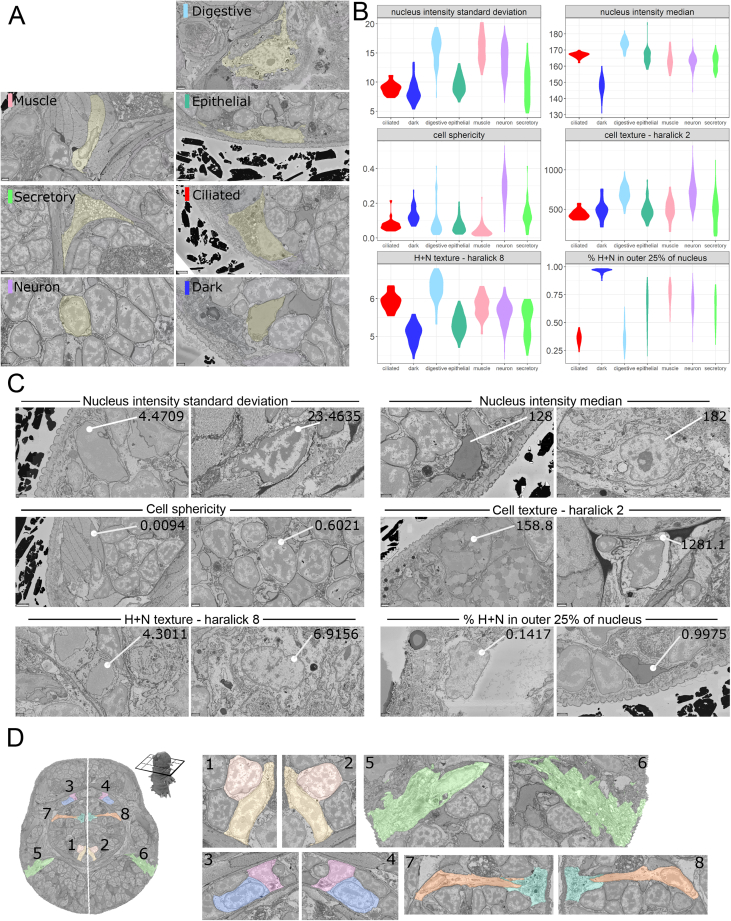
Morphological analysis of cell types and bilateral pair analysis, related to Figure 3 A: Examples of manually identified cell types from the EM dataset. B: More examples of morphological features that vary between cell types (extension of Figure 3D) C: Example images from the EM dataset of cells with low and high values for a selection of morphological features. The morphological features shown here are the same as in B. D: Higher resolution images of the example bilateral pairs from Figure 3G. Numbers indicate the location of the images in the overview on the left. Panels A, C and D are available as bookmarks in the PlatyBrowser.

**Figure 3:**
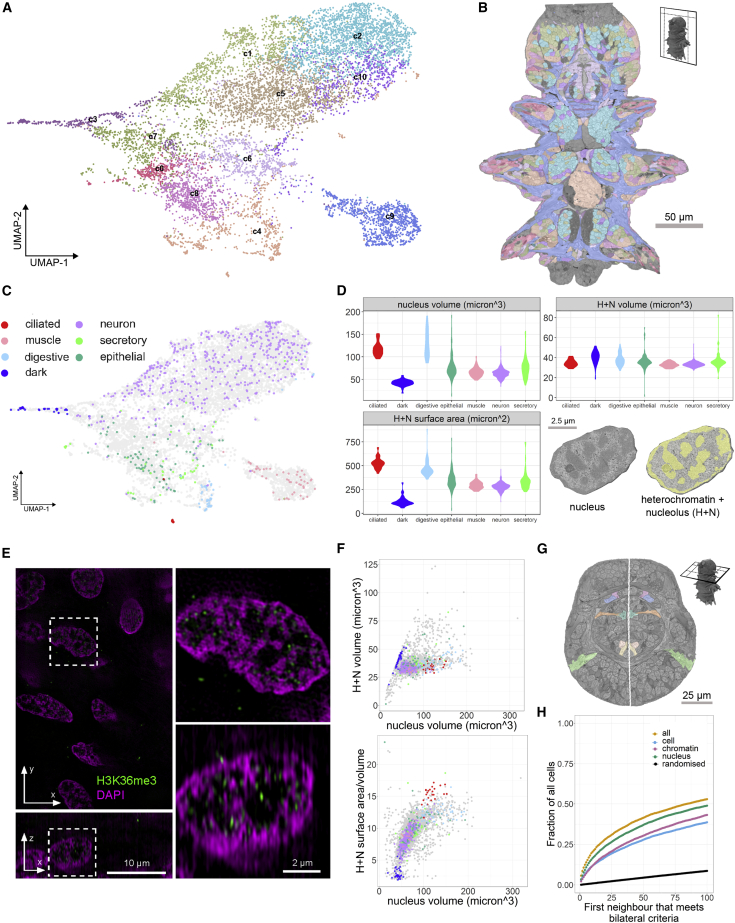
Morphological clustering of segmented cells (A) UMAP of all cells based on morphological features, colored by morphological clusters (c0–c10). (B) Morphological clusters mapped on an EM section. (C) 978 manually identified cells mapped on UMAP. (D) Violin plots comparing 3 morphological features between the manually identified cell classes. Bottom right: example nucleus with chromatin segmentation. (E) Super-resolution 3D structured illumination microscopy of *Platynereis* nuclei. Immuno-labeled histone H3K36me3 (green) indicates active gene bodies on the surfaces of chromatin domains labeled with 4’, 6-diamidino-2-phenylindole (DAPI) (magenta). Upper panel shows lateral, lower panel orthogonal cross-section of a 3D image stack. (F) Scatterplots relating morphological features between all cells with manually identified cells labeled as in (C) and (D). (G) Example section with 7 bilateral cell pairs (midline in white). (H) Fraction of cells finding potential bilateral partners within a certain number of morphological neighbors (see STAR Methods). Legend refers to the features used. (B) and (G) are available as bookmarks in the PlatyBrowser. See also Figure S2.

The whole-body, cellular-scale segmentation enabled quantitative morphological comparison of all cells. For this, we defined and measured 140 descriptors for cells and nuclei, including chromatin distribution (see Table S1; Figure S2C). We then clustered all cells using a graph-based approach (Figure 3A; see STAR Methods). Mapping clusters to the EM showed that they largely corresponded to neurons, muscles, and epithelial, digestive, or ciliated cells (Figure 3B). We also found a conspicuous group of cells with dark (highly scattering) nuclei and cytoplasm, sparsely distributed in the head. To validate the identity of the morphological clusters, we manually classified 978 cells (Figure S2A; see STAR Methods). These cell classes occupy distinct regions in the UMAP, demonstrating that they can be identified by the morphological descriptors. The exception is the broad category of secretory cells that likely comprise subclasses with a wide range of morphologies (Figure 3C).

We then focused on morphological features characterizing the manually identified cell classes (Figures 3D and S2B). For example, ciliated and digestive cells have larger nuclei than other cells, which goes in concert with a larger heterochromatin surface area (including nucleolus; Figure 3D). In contrast, the heterochromatin volume is similar to that of other cells, in line with our observation that, in *Platynereis* nuclei, the electron-scattering “heterochromatin” represents the actual DNA (with constant volume; Figures 3D and 3E). These relationships also apply to all nuclei (Figure 3F). The larger heterochromatin surface should reflect an increased exposure (i.e., unpacking) of the DNA, which we speculated might be indicative of a higher number of activated genes. Supporting this, active gene bodies (Miron et al., 2020) are found on the heterochromatin surface in *Platynereis* nuclei (Figure 3E). This indicates that, in the 6-dpf young worm, nucleus size is a proxy for the extent of gene activation in a cell type. For the ciliated and digestive cells, the increased number of active genes might relate to the production of cilia and microvilli.

We next explored the power of morphometry with regard to bilateral cell pairs. In the bilaterally symmetrical *Platynereis* 6-dpf young worm, there are hundreds of cell types comprising bilateral homologs (Vergara et al., 2017). For each cell, we ranked all other cells by distance in morphology space and identified potential bilateral partner cells by their mirror-image position (see STAR Methods; Figures 3G and S2D). Based on all morphology features combined, 14% of cells found a potential bilateral partner within the 5 nearest neighbors (20% within 10 and 28% within 20 nearest neighbors; Figure 3H). This is remarkable, given that there are more than 10,000 possible partners and that neurons, which constitute the majority of the cells, are morphologically very similar (Figure 3A). Although all features combined performed the best, followed by nuclear features (shape, intensity, texture, and chromatin distribution), we noted that the chromatin features alone (intensity and texture) still found bilateral pairs with surprising efficiency (e.g., 7% of cells finding a partner within the 5 nearest neighbors, 12% within 10, and 18% within 20). Because bilateral pairs of cells in the 6-dpf *Platynereis* are also identified via identical expression profiles (Vergara et al., 2017), these results suggest some correlation between chromatin morphology and gene expression profile.

### Registration of EM volume and ProSPr gene expression atlas

**Figure S3:**
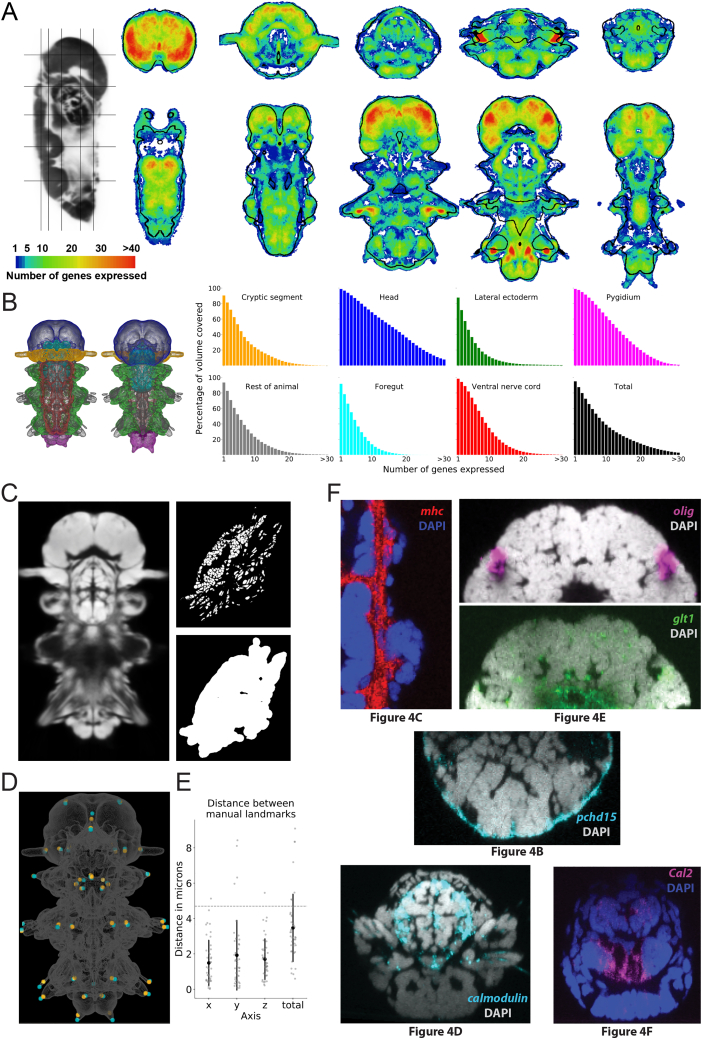
ProSPr expression atlas gene coverage and registration to the EM, related to Figure 4 A: Genetic coverage throughout the animal. Transversal and horizontal sections color-coded based on the amount of expression information, in gene number, for every pixel. Black contours outline the DAPI-based reference, thresholded for illustration purposes. B: Quantification of gene coverage by animal region. Animal regions are colored as indicated in the 3D views. Histograms represent the percentage of volume containing signal for the number of genes indicated in the x axis. (C): Exemplary single planes of the image data stacks, which were used as input to the registration. Left: DAPI: Average DAPI signal of 153 images from the ProSPr atlas. Top-right: EM-Nuclei: Mask of segmented nuclei of the EM individual. Bottom-right: EM-Mask: Binary mask, created by dilation and binarisation of the EM-Nuclei image. The EM-Mask was used to restrict the elastix optimization algorithm to relevant parts of the image. (D): three-dimensional visualization of the overlay after final registration (see methods) between the 43 manually selected landmarks in both datasets. Landmarks in the SBEM dataset are plotted in orange, and landmarks in the ProSPr atlas are plotted in cyan. Spheres have a diameter of 5 μm. Plotted in ProSPr space. The gray outline is an arbitrary mask extracted from the DAPI signal. (E): quantification of the distance between the 43 landmarks in each axis (ProSPr atlas space: ‘x’ corresponds to medio-lateral, ‘y’ to anterior-posterior, and ‘z’ to dorso-ventral axis), and in total. Horizontal dashed line represents the average cell diameter. (F): confocal slices of individual whole-mount *in situ* hybridizations showing raw gene expression for some genes shown in Figure 4.

**Figure 4:**
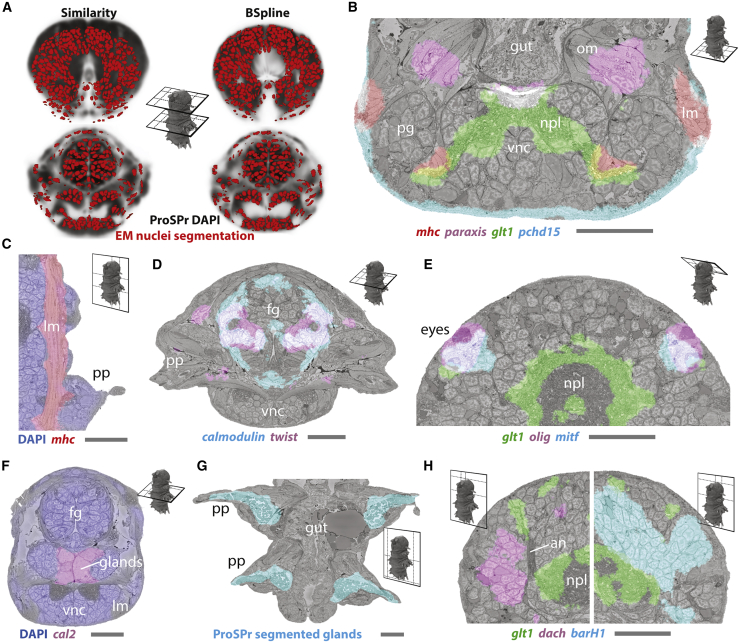
Registration of EM volume and ProSPr gene expression atlas (A) Comparison between similarity and BSpline transform, illustrated with two transversal slices. ProSPr DAPI reference (gray tones) with overlaid segmented EM nuclei (red). Cross-sections through head (top) and foregut (bottom). (B–H) Examples of ProSPr atlas-volume EM overlay. Segmented glands (G) were extracted from the ProSPr dataset using autofluorescence. Scale bar is 25 μm in all images. Raw data for some genes are shown in Figure S3F. (B)–(H) bookmarks are available in the PlatyBrowser. an, antennal nerve; fg, foregut; lm, longitudinal muscles; npl, neuropil; om, oblique muscles; pg, peripheral ganglia; pp, parapodia; vnc, ventral nerve cord. See also Figure S3.

The high stereotypy of *Platynereis* development (Vopalensky et al., 2019) allows for the generation of whole-body gene expression atlases with cellular resolution. This is achieved by whole-mount *in situ* hybridization, DNA-staining-based image registration to a common reference, and profiling by signal probability mapping (ProSPr) (Achim et al., 2018; Asadulina et al., 2012; Vergara et al., 2017). We expanded the 6-dpf ProSPr atlas for an improved representation of expression profiles throughout the body (Figures S3A and S3B; see STAR Methods), achieving a coverage of >7 genes in half of the cells. We next set out to integrate the SBEM dataset with the ProSPr atlas. Stepwise image registration (see STAR Methods) yielded a registration accuracy below one cell diameter (<4.7 μm; Figures 4A and S3E), allowing the overlay of gene expression and ultrastructural data.

The mapping of tissue markers confirmed the registration accuracy. For example, muscle markers label myocytes, with *myosin heavy chain* (*mhc*) in longitudinal and other muscles (Figures 4B and 4C) and the *paraxis* and *twist* transcription factors in oblique muscles (Figure 4B) and stomodaeal muscles (Figure 4D), respectively. We also find neural transcripts, such as the glutamate transporter *glt1* (Figures 4B, 4E, and 4H) and the nicotinic acetylcholine receptor (*nAchR*), accurately overlapping the neuropil, indicating that these transcripts are transported into neurites. Epithelial genes, such as the cadherin *pcdh15* (Figure 4B), the metabotropic glutamate receptor *grm7*, and the serine protease neurotrypsin *ntrps*, overlap with the flat epithelial cells lining the body (Achim et al., 2018) and the cathepsin-like L-protease *cal2* with oral gland cells (Figure 4F) that resemble salivary glands in other annelids (Walz et al., 1988). Other expression patterns appear to obey tissue boundaries within an organ, such as the calcium-binding *calmodulin* in the stomodeum (Figure 4D) and the neuronal transcription factors *dachshund* (*dash*) and *barH1* in nervous tissue (Figure 4H). Identifiable structures overlap between the two datasets (Figure 4G). Finally, we used the adult eyes for registration validation, with their complex cellular morphology comprising rhabdomeric photoreceptor cells and pigment cells (Figure 1C; Rhode, 1992), and revealed distinct patterns for the basic-helix-loop-helix (bHLH) transcription factor *mitf* demarcating pigment and photoreceptor and the bHLH factor *olig* demarcating photoreceptor cells alone (Figure 4E).

### Correlation of gene expression with morphologically defined tissues

**Figure 5:**
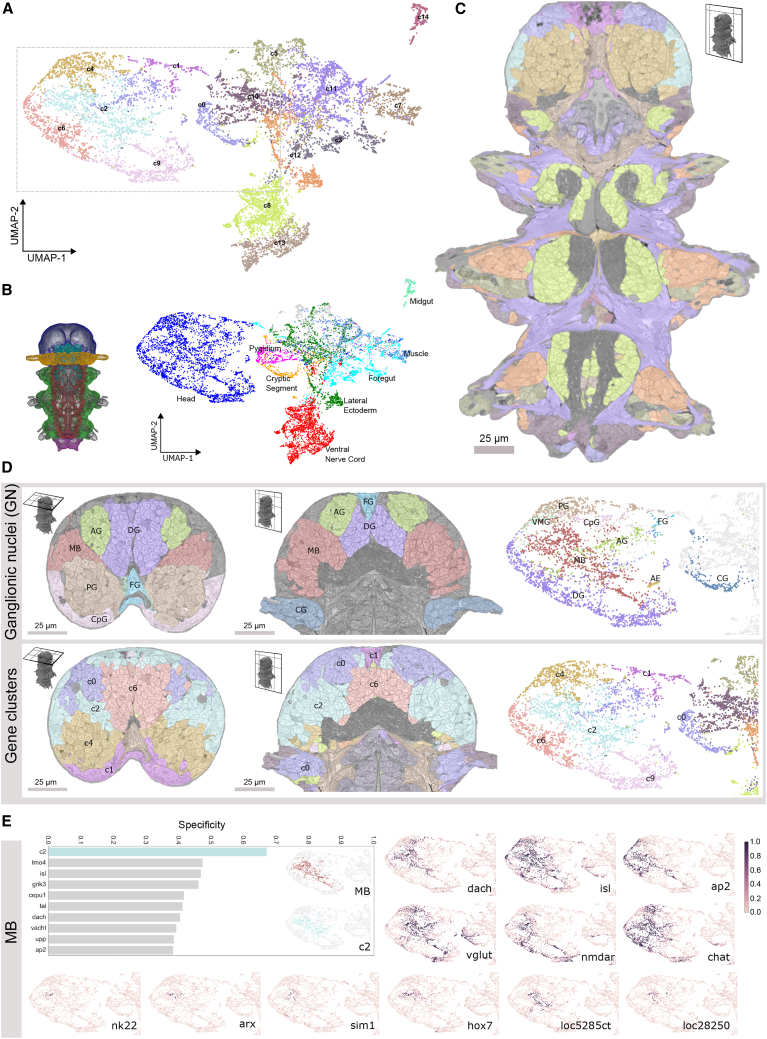
Correlation of gene expression with morphologically defined tissues (A) UMAP of all cells based on the expression of 201 genes. Points are colored by gene expression clusters (c0–c14). Gray rectangle, part of the UMAP shown in (D) and (E). (B) Main body parts in 6-dpf *Platynereis* with matching colors between animal regions and UMAP. (C) Gene clusters mapped onto an EM section. (D) Comparison of anatomically (top) and genetically (bottom) defined units mapped on transversal (left) and horizontal (middle) head section and UMAP (right). (E) F1 specificity score for mushroom bodies (MBs) and the 10 top scoring genes and clusters (see STAR Methods). The MB, cluster c2, and the expression of 12 genes mapped onto the head region of the UMAP are shown. See Figure S4 for specificity of the remaining ganglionic nuclei (GN). For (C) and the EM overlays of (D), bookmarks are available in the PlatyBrowser. AEs, adult eyes; AG, antennal GN; CG, cirral GN; CpG, circumpalpal GN; DG, dorsal GN; FG, frontal GN; MBs, mushroom bodies; PG, palpal GN; VMG, ventro-medial GN. See also Figure S4.

The unique combination of cellularly resolved gene expression and ultrastructure allowed us to explore the interplay between differential gene expression and morphology across all body cells. To do so, an expression value was calculated for every gene in every segmented cell as the fraction of the cell’s volume overlapping the registered gene expression volume (referred to as “overlap assignment”). We then used a graph-based clustering approach (with visualization by UMAP) that resulted in 15 clusters (Figure 5A). To understand how these clusters relate to the animal’s anatomy, we mapped body parts (head, cryptic segment, ventral nerve cord, pygidium, lateral ectoderm, foregut, and midgut) onto the UMAP and found a strict correlation with gross anatomy, with six genetic clusters spanning the head and two the ventral nerve cord (Figure 5B). Mapping these clusters onto the EM volume revealed that segmented cells that belong to the same genetic cluster occupy spatially coherent territories in the body, with a clear correspondence of genetic and tissue boundaries (Figure 5C). In the trunk, these transcriptionally defined domains correspond to ventral nerve cord cells, peripheral ganglia, musculature, and gland cells.

**Figure S4:**
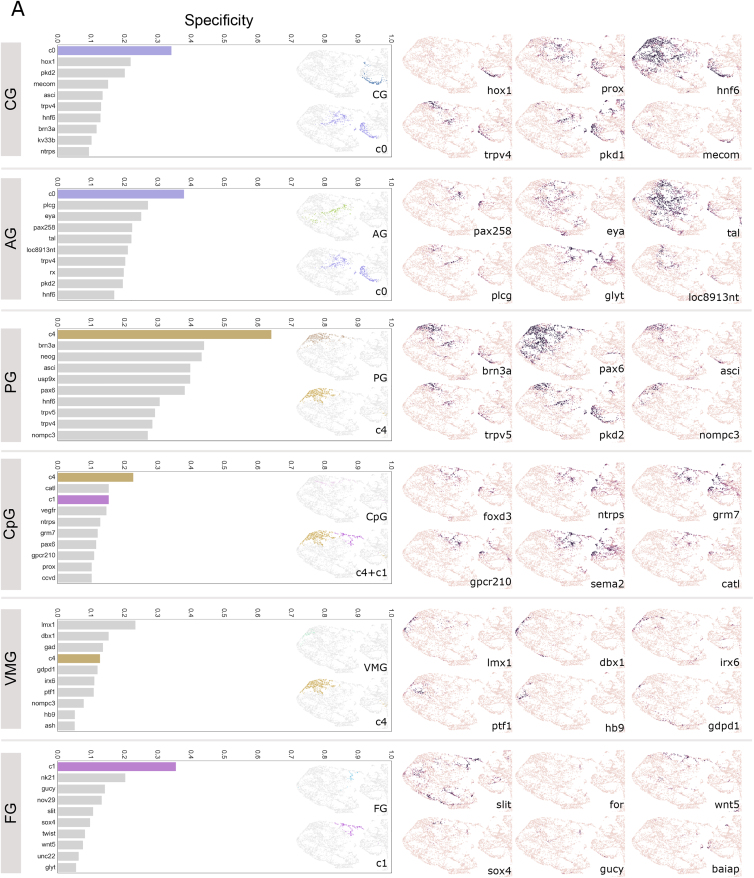

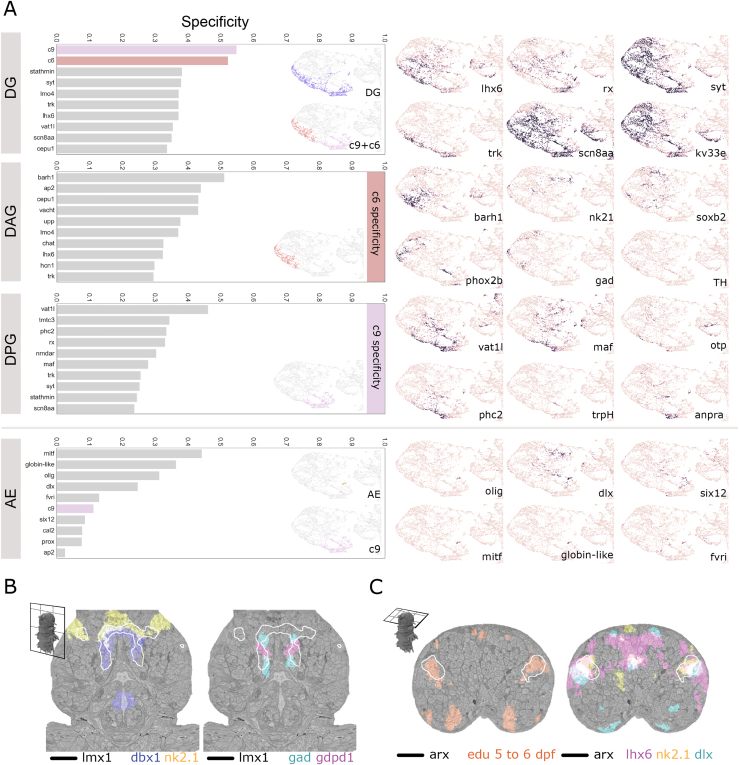
Specificity of gene clusters and individual genes for head ganglia, related to Figure 5 A: Comparison of specificity of gene clusters and individual genes for the head ganglia. Left column – graphs of top 10 scoring genes (gray bars) or gene clusters (colored bars) by F1 specificity score (see methods). Inset– zooms of the head region of the UMAP from Figure 5A colored by ganglia (top) and top scoring genetic clusters (bottom). Right column - gene expression overlap value (0-1, same scale as in Figure 5E) for example genes. Note: here we show only the head region of the UMAPs, for easier comparison, but some genes and gene clusters have expression domains outside of the head which contribute to their lower specificity scores. *loc8913nt* corresponds to the ionotropic glutamate receptor *igluR*. Other genes starting with ‘loc’ are GPCR-related but without clearly identified homologs. Abbreviations: AG, antennal ganglia; CG, cirral ganglia; CpG, circumpalpal ganglia; FG, frontal ganglion; VMG, ventro-medial ganglia. B: Frontal cross-section of the ventral brain illustrating co-expression of genes in the ventromedial ganglion. The expression region of *lmx1* is illustrated with white contours. C: Dorsal cross-section of the brain illustrating one of the proliferative regions of the mushroom bodies and the specific co-expression of transcription factors. The expression region of *arx* is illustrated with white contours. Panels B and C are available as bookmarks in the PlatyBrowser. Abbreviations: AE, adult eyes; DG, dorsal ganglion; DAG, dorso-anterior ganglion; DPG, dorso-posterior ganglion.

For a more detailed analysis of how gene expression relates to tissue boundaries, we focused on the animal’s head (Figure 5D)—the region with the highest gene density in the expression atlas (Figure S3A) and a more refined subdivision in the UMAP (Figure 5A). The 6-dpf brain is largely differentiated (Chartier et al., 2018) with discernible anatomical subunits called ganglionic nuclei (GN) (Engelhardt et al., 1982) that we manually segmented from the EM volume (Figure 5D, upper row). We then noted that the 6 head clusters closely matched the anatomically defined GN (Figure 5D). To quantify this, we calculated a specificity score for each genetic cluster and gene, for every ganglion (Figures 5E and S4A; see STAR Methods). Remarkably, for almost all GN, specificity values for individual genes were considerably lower than those for gene clusters. Notably, genes with relatively high specificity values often encoded transcription factors, such as *dach*, *isl*, or *ap2* (Figure 5E), whose expression often occurred in coherent and overlapping domains. Highly specifically expressed transcription factors were found in subsets of one GN, as observed for *arx*, *hox7*, *nk2.2*, and *sim1* (Figure 5E). These findings indicate that the *Platynereis* 6-dpf head is subdivided into transcriptional domains that are defined by the combined expression of several transcription factors largely corresponding to GN. Notably, the two clusters c6 and c9 together corresponded to a single GN, with the c6 cells mapping strictly anteriorly and the c9 cells strictly posteriorly. We thus subdivided that unit into a dorso-anterior and a dorso-posterior GN for subsequent analysis.

### Expression profile and neuropil projections of head ganglia

**Figure 6:**
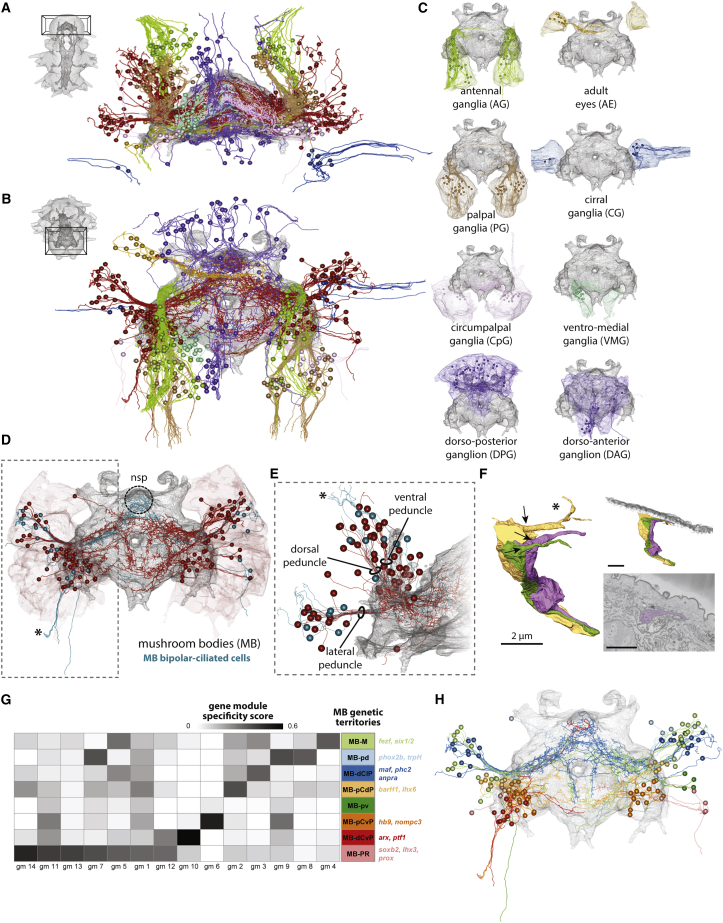
Expression profile and neural projections of head ganglia and mushroom bodies (A) Ventral 3D visualization of all neurons reconstructed in this study, with cell bodies represented as spheres and cellular projections skeletonized. Neuropil is represented as a gray mesh, and neurons are colored as their GN in Figure 5. (B) Same as in (A) from an anterior-dorsal view. (C) Anterior-dorsal 3D views of individual GN illustrating the volume of all constituting cells and the traced cells. (D) Same as in (C) for the MB, highlighting bipolar cells in cyan. (E) Frontal view of the right mushroom body (dotted box in D). Rings indicate the dorsal and ventral peduncles of the mushroom bodies (see Figure S5). (F) Dense reconstruction of sensory endings of 3 colored bipolar cells (asterisks in D and E). Arrows indicate the base of the sensory cilia. On the top right, same rendering from a different perspective to show that the cilia remain below the cuticle, rendered in gray. On the bottom right, EM image for one of the cilia. (F) is available as a bookmark in the PlatyBrowser. (G) Heatmap showing the specificity score of gene modules (gm) for the different genetic territories in the MB (see Figure S5 and STAR Methods). (H) MB traced cells colored by the genetic territory (G and Figures S5B–S5E) they belong to. MB-M, mantle; MB-pd, posterior-dorsal; MB-dClP, distal calyx lateral peduncle; MB-pCdP, proximal calyx dorsal peduncle; MB-pv, posterior-ventral; MB-pCvP, proximal calyx ventral peduncle; MB-dCvP, distal calyx ventral peduncle; MB-PR, progenitor region. See also Figure S5.

The correspondence of transcriptional domains and GN in the head suggested distinct functions and projections of these units. In *Platynereis*, neuronal cell bodies surround a central neuropil, where they project and form synapses (Engelhardt et al., 1982; Verasztó et al., 2020). We pseudo-randomly selected 384 neurons in the head, manually traced their arborizations (see STAR Methods), and observed unique and distinct neurite projections and innervation fields for the neurons of each GN (Figures 6A–6C). This allowed us to define sensory and central GN of the 6-dpf brain based on gene expression, location, neuronal morphology, and projection information.

The palpal, antennal, and cirral GN (PG, AG, and CG), associated with the prominent sensory appendages of the head, contained many bipolar sensory neurons (89%, 88%, and 83%). The sensory endings of these cells enter the respective appendage, and their axons form prominent tracts that project locally in the central neuropil, with few axons crossing the midline in a single commissure (Figure 6C). We found many genes specifically expressed in two or all three sensory GN (in line with AG and CG belonging to one single cluster—c0), including the transcription factors *brn3*, *prox*, and *asci.* This indicates that these GN and their appendages are serial homologs (see Discussion). Other transcription factors were expressed in one of the three sensory ganglia only (e.g., *hox1* in CG, *pax258* in AG, and *pax6* in PG), reflecting their divergent fates. In addition, the sensory ganglia jointly expressed the receptors *pkd1* and *pkd2*, the Trp channels *trpV4* and *trpV5*, and the ionotropic glutamate receptor *iGluR* (Figure S4), consistent with neurons in all three ganglia responding to glutamate (Chartier et al., 2018) and indicating that they also detect mechanical stimuli (Bezares-Calderón et al., 2018). Next, we inspected the circumpalpal GN (CpG) and found that they represented epithelial sheaths surrounding the PG with 26% bipolar sensory neurons. In line with epidermal properties, we found expression of markers, such as neurotrypsin (*ntrps*) and metabotropic glutamate receptor7 (*grm7*) (Achim et al., 2018).

We next identified the dorso-anterior and ventromedial GN (DAG and VMG), enriched in interneurons and with fewer bipolar sensory neurons (24% and 0%, respectively). DAG encompasses the medial tip of the brain with a dorsal *barH1*+ and a ventral *nkx2.1+*, *dbx1+*, *lmx1+* part, both of which contain GABAergic neurons (Figure S4). The paired VMG encompass *dbx1+*, *lmx1+*, *gdpd1+* GABAergic interneurons ventrally adjacent to the DAG (Figures S4A and S4B). Both VMG and DAG neurons project to the ventral-most portion of the neuropil, with VMG neurons sharing their projection target area with CpG neurons (Figure 6C).

The large dorsoposterior GN (DPG) forms a dorsal cap to the remainder of the brain. 50% of the cells are bipolar sensory and innervate the neurosecretory plexus (nsp), a distinct neuropil region formed by projections from peptidergic sensory-neurosecretory cells (Tessmar-Raible et al., 2007) filled with dense core vesicles and forming very few synapses (Williams et al., 2017). This unique brain part has been previously referred to as apical nervous system (Achim et al., 2018; Tosches and Arendt, 2013; Williams et al., 2017) and has been homologized to neuroendocrine pars intercerebralis of insects and associated central complex neuropil (He et al., 2019). Consistent with this, we detect a unique transcription factor signature (*vat1-like*, *maf*, *otp*, and *rx*; Achim et al., 2018) and neurosecretory markers, such as the prohormone convertase *phc2*, the serotonin-synthesizing enzyme tryptophan hydroxylase *trpH*, and the peptide receptor *anpra* (Figure S4). Laterally abutting the DPG, we found the adult eyes (AEs) specifically expressing the bHLH transcription factors *olig* and *mitf* (see above) and a photoreceptor marker, the neuropeptide *fvri* (Jékely et al., 2008). Our tracings confirmed previous findings for 3-day-old worms showing that the AE projections enter a commissure ventral to nsp (Williams et al., 2017; Figure 6C). Only cells from one other GN project to the nsp, those from the mushroom bodies (MBs).

### Cellular architecture and molecular anatomy of the MBs

**Figure S5:**
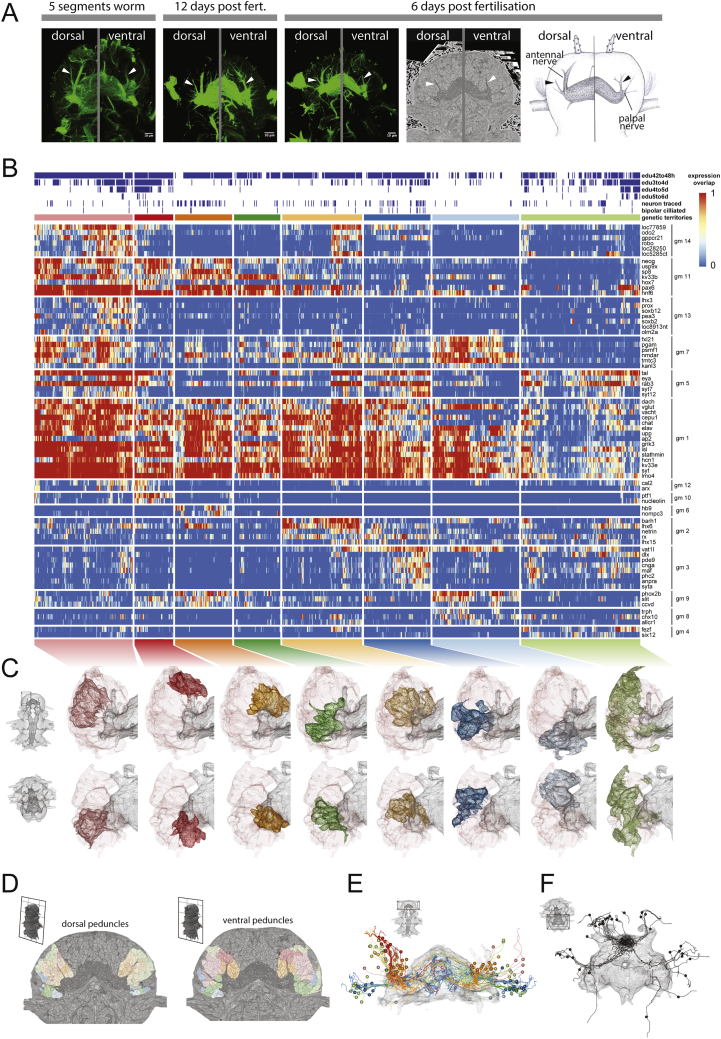
Anatomico-molecular analysis of the mushroom bodies at 6 dpf, related to Figure 6 A: Illustration of the identification of mushroom bodies peduncles from the juvenile worm, where both the cellular and neuropil structures are clearly recognizable, to the 6 dpf *Platynereis*. The first three panels are maximum projection images of confocal stacks, obtained from acetylated tubulin immunostainings. The fourth panel is obtained from the EM dataset. The fifth panel is a simplified illustration of the relevant structures shown in the rest of the panels. All panels are composed of two views at different dorso-ventral locations to highlight the dorsal and ventral peduncles, indicated with arrowheads. B: Heatmap for all cells that constitute the mushroom bodies (MB) ganglia. Only the 75 most variable genes are shown. Cells are grouped into clusters (genetic territories; see methods), and hierarchically ordered within each cluster. The order of the MB clusters in the heatmap is established using hierarchical clustering of cluster expression means. Rows are ordered by gene modules as in Figure 6G. Genes within each module are ordered by specificity values of each gene (see methods). On the top of the heatmap, blue indicates cells positive for proliferative EdU stainings done at distinct developmental stages, as well as cells that are traced, and those found to show bipolar projections (sensory endings). Genes starting with ‘loc’ are GPCR-related but without clearly identified homologs. C. 3D views of the MB genetic territories, each color-coded according to their colors in the heatmap. The neuropil is plotted in gray, and the mesh of the entire mushroom body ganglion is plotted as well for reference. The top row shows the ventral view and the bottom row the anterio-dorsal view. The regions shown are specified in the models shown in the left. D. EM slices at the level of the two peduncles. E. Frontal 3D view of the cells traced for the mushroom bodies ganglia (see Figure 6G). In D and E, cells are color-coded according to which MB genetic territory they belong to. F. Anterio-dorsal 3D view of the traced dark cells (see section on cell morphology; Figures 3 and S2).

The tracings for the MBs revealed three distinct axon bundles projecting to the neuropil (Figures 6D and 6E). Two of these bundles are easily traced by immunostaining through development and correspond to the dorsal and ventral peduncles of the adult MBs (Figure S5A; Dürichen, 2016; Tomer et al., 2010). At 6 dpf, these dorsal and ventral peduncles are relatively close to each other and oriented parallel to the anterior-posterior axis of the animal. A third prominent peduncle of unknown fate is oriented laterally and perpendicular to the dorsal and ventral peduncles (Figures 6D and 6E).

Unexpectedly, we found that 17% of traced MB cells represented bipolar sensory neurons, which indicated that the 6-dpf *Platynereis* MBs have sensory properties. We noted that the dendrites of some of these neurons coalesce in tight bundles, so as to form joint sensory structures (asterisk in Figures 6D and 6E). We densely reconstructed the distal endings of the sensory bundles and found terminal cilia that do not penetrate the cuticle (Figure 6F), as characteristic for uniciliate non-penetrative sensory neurons (Purschke, 2005). We also observed that, among the MB cells, the bipolar sensory neurons were the only ones projecting to the nsp (Figure 6D), very similar to the sensory-neurosecretory cells of the apical nervous system. Intriguingly, these nsp-projecting bipolar sensory cells turned out to be the dark cells (Figures 3C, 3D, and S2A–S2D; see above). We accordingly examined all traced dark cells of the 6-dpf brain and found that they were mostly located on the DPG and projected specifically to the nsp (Figure S5F). This established a strong link between MBs and DPG, which both contain dark bipolar sensory-neurosecretory neurons projecting to the nsp.

Next, we investigated the molecular anatomy of the MB neurons (Figures 6G and S5B–S5D). We found eight genetically defined territories and 14 gene modules differentially expressed in these territories (see STAR Methods). Consistent with what we had observed for the entire brain, the genetically defined MB sub-territories corresponded to spatially and morphologically coherent groups of cells in the context of MBs anatomy. Several groups of neurons jointly fed into the ventral, dorsal, or lateral peduncles (Figures 6H and S5C–S5E) and thus constituted proximal or distal portions of the MB calyces. Each of these groups expressed distinct sets of transcription factors (e.g., *arx* and *ptf1* in dCvP, *hb9* in pCvP, *barh1* in pCdP, and *dlx* and *maf* in the dClP) and marker genes for neurosecretion (e.g., *phc2* and *anpra* in dClP), suggesting affinities of this part of the MBs to the apical nervous system (Figures 6G and S5B). Neurons of each territory also showed distinct projection areas into the central neuropil (Figures 6H and S5E).

We found that dCvP, together with two additional territories (MB-PR and MB-M), were enriched in cells labeled by our EdU proliferation assays (Figure S5B) that are devoid of long neuronal processes (see STAR Methods). The proliferating cells within these regions were located peripherally, close to the surface. These territories are identified as MB progenitor regions by their unique expression of two *soxb2* paralogs and the specific co-expression of the transcription factors *nkx2.1*, *dlx*, *arx*, and *lhx6* (Figure S4C). In addition, MB-PR and MB-M express many genes shared exclusively with other selected territories (e.g., gene modules 2, 3, 5, 7, 11, and 14), consistent with the notion that they contain diverse MB progenitors.

### Assigning virtual cells to segmented cells

**Figure 7:**
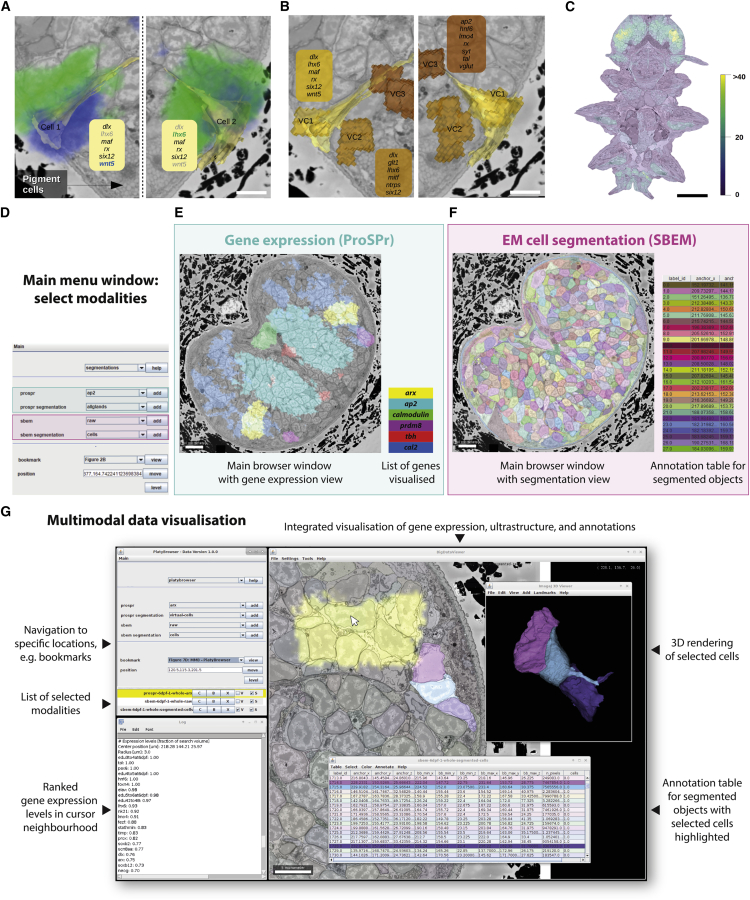
Virtual cells and the PlatyBrowser (A) Assignment by gene overlap illustrated for *lhx6* and *wnt5* (blue and green), for a pair of bilateral cells. In boxes, assigned genes show >50% overlap. Genes in light gray fail to be assigned. Scale bars: 5 μm. (B) Assignment to virtual cells for the same cells as in (A). (C) Number of assigned genes per segmented cells after virtual cell assignment (scale bar: 50 μm). For (A)–(C), bookmarks are available in the PlatyBrowser. (D) User interface to select image sources, change their appearance, and navigate to specific locations in the animal. (E) BigDataViewer, showing the SBEM image in a region of the adult head, with the ProSPr signal for six different genes. (F) BigDataViewer of the same section as in (B), now displaying the cellular segmentation. (G) Screenshot of the PlatyBrowser illustrating the integration of modalities and additional functionalities: the expression of gene *arx* is shown in yellow; three segmented neurons are shown next to it; and annotation table below with highlighted rows that correspond to selected objects. The 3D Viewer window shows a rendering of the selected cells; the colors for a given object are identical in the 2D Viewer overlay, 3D rendering, and table. Below the main menu, the log window shows a ranked list of gene expression where the mouse cursor is positioned (white arrow). See also Figure S6.

**Figure S6:**
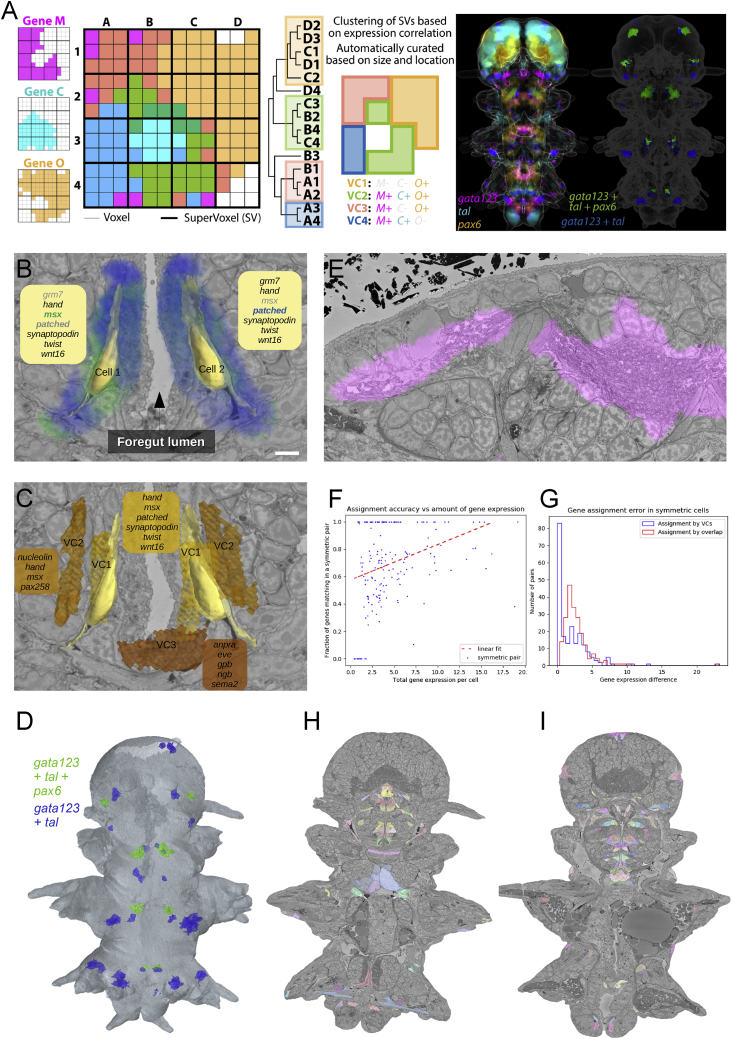
Gene expression assignment, related to Figure 7 A: Generation of Virtual Cells. On the left, hypothetical spatial expression of three different genes in a 12 × 12 voxel array. In the matrices, thin lines demarcate the voxels and dark ones supervoxels. Voxel colors indicate all possible combinations of expressions (e.g., gene C + gene M in dark blue). The hierarchical tree illustrates the process of clustering supervoxels based on expression information, which renders groups of supervoxels called Virtual Cells (VCs), that are then automatically curated based on size and spatial location. The VCs are visualized spatially on the matrix next to the tree with their expression information. On the right, illustration of this procedure with real data. Three genes are shown on a projection image of the full dataset using similar coloring for the co-expression as in the example on the left. Next to it, the coloring of VCs showing a specific expression pattern (2 examples). Note that each of these groups is composed of many VCs but all have the same coloring for illustration purposes. B-C: The difference between assignment by overlap and assignment to genetically nearest Virtual Cell. (B): Assignment by overlap: biological variability and registration error resulted in slight asymmetry of the gene expression volumes of the genes *patched* and *msx* (blue and green). Gene lists correspond to genes that would be assigned as expressed in the cell if the assignment was done by volume overlap (regular print for genes with > 50% overlap, light gray font for the rest). With a fairly conservative 50% overlap threshold, the resulting assignment for the bilaterally symmetric cells would be different (scale bars: 5 μm). (C): Assignment to Virtual Cells: for each segmented cell the neighboring Virtual Cell that has the smallest genetic difference is assigned. This results in a consistent assignment of denoised genetic profiles (scale bars: 5 μm). (D): The assignment of the Virtual Cells shown on panel A to the segmented cells. The cells in blue were assigned Virtual Cells that express genes *gata123* and *tal*, the ones in green - genes *gata123*, *tal*, and *pax6*. (E): ‘Gene leaking’ of *glt1* (glutamate receptor). While the true expression is confined to the neuropil, the bordering regions such as neural somas, muscles and epithelial cells also show a high level of expression, originating from sample variability and limited registration accuracy. F: Dependency of the assignment accuracy on the total level of gene expression in a cell (the fractions of gene expression for each gene summed up). The assignment performed better for the cells in gene rich areas. (G): Assignment errors in the symmetric cells pairs for the Virtual Cells assignment and assignment by overlap. Assignment error is defined as the absolute difference in gene expressions assigned to the cells of a symmetric pair. H-I: Examples of symmetric cell pairs – cells with similar mirror location and morphology, supposedly representing the same cell type and expressing the same genes. For panels B,C,H and I bookmarks are available in the PlatyBrowser.

At single-cell resolution, assignment of gene expression by overlap may result in asymmetric profiles (Figures 7A and S6B), due to biological variability of the specimen, deformations during sample preparation, and registration error (Figure S6E). To overcome this, we first identified spatially coherent units of homogeneous gene expression in the ProSPr atlas that we named virtual cells (VCs) (Figure S6A; see STAR Methods). In total, we found 12,393 VCs, a number very similar to that of EM-segmented cells (11,402). We then assigned VCs to single segmented cells on each side according to minimal expression difference (Figures 7B and S6C; see STAR Methods). To benchmark our method, we compared the accuracy of overlap versus VC assignment by testing symmetric cell pairs (Figures S6H and S6I) for identical gene expression profiles (Vergara et al., 2017). VC assignment resulted in a mean discrepancy of 2.0 genes per cell pair, performing ∼30% better than overlap assignment with 2.8 genes mean discrepancy (Figure S6G). Specifically, VC assignment performed better in ∼70% of all pairs. Reflecting the atlas coverage (Figures S3A and S3B), VC gene density was not uniform throughout the animal body (Figure 7C). We observed that the assignment quality is higher for cells with better expression coverage (Figure S6F) and thus expect that adding more genes to the atlas will further improve gene assignment at cellular resolution.

### PlatyBrowser: Multimodal image data exploration for *Platynereis dumerilii*

The registered ProSPr and SBEM datasets form a valuable resource containing rich biological information. However, the size of this resource (currently 231 image sources adding up to 2.02 TB lossless compressed image data) poses a challenge to its effective interrogation for scientific discovery. We therefore developed MoBIE: an open-source platform for multimodal big image data exploration and sharing. MoBIE consists of an object store backend for cloud-based image data hosting (Bogovic et al., 2020) and a Fiji (Schindelin et al., 2012) plugin for interactive image and segmentation data browsing. We use MoBIE to deploy the “PlatyBrowser” (Figures 7D–7G). Thanks to lazy loading (Pietzsch et al., 2015) from a public object store, the PlatyBrowser provides interactive access to the complete TB-sized *Platynereis* dataset to everyone with a standard computer and an internet connection (see STAR Methods).

## Discussion

### Seeking multimodality

A comprehensive understanding of cell types and their role in animal development, evolution, and physiology requires the integration of distinct modalities (Bates et al., 2019; Hobert et al., 2016; Nicovich et al., 2019). In particular, correlating gene expression with cellular and subcellular morphology is crucial to understand the principles that guide the decoding of expression information into cellular phenotypes.

One direction has been to combine expression profiling with cellular imaging. Several recent studies have implemented this for restricted sets of cells. For example, retrograde labeling of neurons prior to sequencing allows integration of partial projection information with transcriptomics (Kim et al., 2019; Phillips et al., 2019; Tasic et al., 2018). Photoconversion of dyes in selected cells can be used to correlate single-cell sequencing with live imaging techniques (Lee et al., 2019; Pfeffer and Beltramo, 2017). Other approaches based on probe hybridization or local sequencing provide expression information *in situ*, recovering the position of the cells interrogated (reviewed in Lein et al., 2017). Such spatial transcriptomics techniques can be correlated and complemented with single-cell transcriptomics (Kim et al., 2019; Phillips et al., 2019; Qian et al., 2020), and recent developments using viral barcoding have also enabled the integration of long-range projection information in a brain-wide manner (Chen et al., 2019). However, these approaches only retrieve multimodal information for small subsets of cells and/or their scalability is not straight-forward beyond a set of tissue slices (Qian et al., 2020).

The other possible direction is to complement EM with expression profiling. So far, such attempts have focused on cell-type populations (Davis et al., 2020) or have relied entirely on prior knowledge about the cells of interest. For example, this link is possible for recognizable neurons, such as mechanoreceptors, nocireceptors, and MB neurons in the *Drosophila* larvae (Ohyama et al., 2015; Saumweber et al., 2018) or peptidergic and ciliomotor neurons and mechanoreceptors in the *Platynereis* larvae (Bezares-Calderón et al., 2018; Verasztó et al., 2017; Williams et al., 2017). This approach can ultimately generate a catalog of functional, molecular, and morphological data for a multitude of cells in an organism, as is the case for the *C. elegans* nervous system with only 302 neurons (Hobert et al., 2016). More recently, proposals for the multimodal description of the *Drosophila* central nervous system, with 5.000 times more neurons than *C. elegans*, have been put forward (Bates et al., 2019). The rationale behind these approaches relies on anatomical information gathered through the use of cell-type-specific genetic markers and/or GAL4 lines. This restricts the use of cell-type integrative approaches, making it hard to scale to the organism level and hard to translate to species in which genetic manipulation is labor intensive. We now push these efforts to a new level by providing the first multimodal atlas combining EM and expression data for an entire animal.

### A fully segmented volume EM dataset of an entire animal

Ultrastructural analysis of entire animals so far involved the manual collection of serial sections (Hall and Altun, 2007; Randel et al., 2014) and targeted manual or semi-automated reconstructions for sparse morphological and phenotypic characterization (Anderson et al., 2011; Bumbarger et al., 2006, 2013; Cardona et al., 2012; Kasthuri et al., 2015; Wanner and Friedrich, 2020; Wanner et al., 2016a; White et al., 1986). More recently, machine-learning-based reconstruction pipelines enabled the segmentation of entire neuronal circuits at synaptic resolution (Heinrich et al., 2018; Januszewski et al., 2018; Scheffer et al., 2020). Adapting SBEM approaches to *Platynereis*, we have imaged a full individual at high resolution (10 × 10 × 25 nm^3^), enabling analyses that span the length scales between the anatomy of entire organs and cellular ultrastructure. Extending existing pipelines (Beier et al., 2017; Pape et al., 2019), we also present the first automated segmentation of individual cellular somata, nuclei, and chromatin for a complete animal imaged in EM. Our method segments the whole multiple terabyte dataset in less than 3 days using at most 600 CPUs on a computer cluster and can be directly applied to similar datasets.

Taking advantage of 140 descriptors for 11,402 segmented cells, we provide a morphometry-based clustering that subdivides the body into cell classes, including neurons and the “dark” sensory-neurosecretory cells. This factors in not only cellular shapes and cytoplasmic texture but also nuclear shapes and chromatin features that reflect the regulatory state of the cells. In this context, we uncover a link between the nuclear size, heterochromatin volume, and regulatory state. This follows the notion that, upon gene activation, the heterochromatin “unfolds”, increasing its surface area so that active transcription takes place at the interface of the two chromatin phases. This morphometric representation of cell-type-specific regulatory states also underlies the power of our resource in detecting bilateral cell pairs (Vergara et al., 2017), which is highly efficient using chromatin morphometric data only.

### Toward a genetic definition of tissue

A tissue is commonly defined as an ensemble of similar cells and their extracellular matrix with a specific function. Classically, identification of tissues relies on similarity in phenotype of the partaking cells as also manifest in our body-wide morphological clustering. Beyond that, our resource uniquely enables the genetic clustering of all segmented cells in the *Platynereis* body. Recent whole-body, single-cell transcriptomics have been the first to provide genetic clustering of all body cells (Achim et al., 2018; Packer et al., 2019; Sebé-Pedrós et al., 2018a, 2018b). In the absence of spatial mapping at late differentiation stages, however, these studies have not yet resolved to what extent, and at what hierarchical level, the genetically defined clusters represent coherent tissues. Our genetic clustering based on 201 differentially expressed genes subdivides the *Platynereis* body into 15 clusters that demarcate distinct tissues and body parts. In the central nervous system, these clusters represent spatially coherent groups of neurons, whose boundaries match those of hand-segmented ganglionic nuclei, which can thus be defined as genetically similar ensembles of cells, termed “transcriptional domains” (Achim et al., 2018). Our data further show that these domains are best defined by the combined expression of several regulatory genes rather than a single gene. This includes homeodomain factors, such as Pax6, that have been implicated in tissue specification (Rungger-Brändle et al., 2010; Zhang et al., 2019). Our neuronal tracing further reveals that the genetically defined ganglionic nuclei comprise neurons with similar projection patterns. In line with this, previous work identified transcription factors that control tissue integrity via the establishment of an “adhesive code”, as shown for Pax6, that controls axonal connectivity (Jones et al., 2002).

Our unique combination of cellular-resolution gene expression, anatomical segmentation, and neurite tracing yields new insights into the overall organization of the body. For example, we provide evidence that the *Platynereis* head is composed of both segmentally iterated and unique components. Solving a long-standing question about the relatedness and significance of annelid head appendages (e.g., Weigert et al., 2014), our data reveal that *Platynereis* antennae, palpae, and peristomial cirri express transcription factors and Trp channels that are shared with segmental trunk appendages. This indicates that the sensory head appendages and associated ganglia are serial duplicates in a segmental “mechanosensory girdle” (Verasztó et al., 2020). Within this girdle, antennae and palpae may represent separate appendages of the same anteriormost segment, as they project to related areas of the anterior neuropil. In a similar manner, the *dbx+*, *lmx1+*, *gad+* neurons in the ventral brain form part of a segmentally iterated series of GABAergic neurons extending to the nerve cord (see bookmark for Figure S4C in the PlatyBrowser). In contrast, the molecular and morphological characteristics of the *Platynereis* dorsal brain do not reoccur in other body regions. For example, the sensory neurons of the DPG are unique in their combined transcription factor identity and in their projection to the neurosecretory plexus (Achim et al., 2018; Tessmar-Raible et al., 2007; Williams et al., 2017).

### Annelid MBs and the evolution of associative centers

The link between cellular gene expression, tracings, and subcellular morphology improves our understanding of the *Platynereis* MBs. Given their overall morphological similarity to the insect MBs, these are commonly considered associative centers of the annelid brain, composed of densely packed interneurons in the calyces that project through the MB stalks (Heuer et al., 2010). In the past decade, a renewed discussion has emerged whether and at what level insect and annelid MBs represent homologous structures and whether there is any evolutionary relatedness to the vertebrate telencephalic cortex (Strausfeld, 2010; Tomer et al., 2010; Wolff and Strausfeld, 2016). Regarding this, our data provide new insights in two exciting directions.

First, we learn that the MB calyces not only comprise unipolar interneurons but also bipolar sensory neurons that project into the neurosecretory plexus, thus qualifying as sensory-neurosecretory cells, and express marker genes for the apical nervous system. Both ciliary morphology and GPCR expression indicate that the *Platynereis* bipolar sensory-neurosecretory cells act as chemosensors. This is intriguing with regard to the evolution of associative centers. Although both insect MBs and vertebrate telencephalic cortex do not contain olfactory sensory neurons themselves, they receive prominent olfactory input and have particularly elaborate control over olfaction-driven behaviors, suggesting that the distinction of odors may have played a pivotal role in the evolution of associative learning. With our new data, the hypothesis emerges that the evolution of associative centers/MBs may have built on a chemosensory organ that gradually acquired associative properties.

Second, the specific combined expression of the transcription factors *nkx2.1*, *dlx*, *lhx6*, and *arx* in the proliferative region of the *Platynereis* MBs—with *arx* being exclusively expressed in this region—is remarkable when compared to vertebrate telencephalic development. Vertebrate orthologs of these transcription factors are implicated in the specification of the telencephalic interneurons emerging and emigrating from the ganglionic eminences (Marsh et al., 2016) and continue to demarcate striatal and cortical interneurons in the adult mouse telencephalon (Zeisel et al., 2018). In particular, the same full combination is present in the striatal cholinergic interneurons that co-transmit glutamate (Kljakic et al., 2017; Zeisel et al., 2018). This is intriguing, given the broad co-occurrence of vesicular acetylcholine and glutamate transporters in the *Platynereis* MBs. Again, our data shed new light on the evolution of associative centers, in that co-transmitting interneurons may represent one of their oldest neuron types, possibly antedating the split of protostome and deuterostome lineages.

### Conclusions and outlook

Our work represents a first step toward a comprehensive interrogation of the relationship between gene expression and subcellular morphology. Our resource will further improve with the addition of new genes and with the mapping of single-cell transcriptomics data into the ProSPr atlas (Achim et al., 2018) and thus onto the segmented EM cells. For this, we can capitalize on the assignment of VCs to segmented cells as an intermediate for combining expression space and morphospace. Next, the segmentation of additional ultrastructural features (e.g., mitochondria and Golgi apparatus) will expand opportunities for genotype-phenotype correlations.

We also envisage that enhanced resolution of new EM datasets registered onto our resource will allow the automated reconstruction of neuronal circuits and mapping of synapses at a larger scale. Notably, a completely traced serial section EM dataset already exists for 3-dpf *Platynereis* (Verasztó et al., 2020; Williams and Jékely, 2019), with few cells already manually linked to expression data (Williams et al., 2017). With our new resource, this can now be extended to integrate connectomics and transcriptomics for an entire animal and for different developmental stages. Additionally, the multimodal capacity of the PlatyBrowser will allow the incorporation of physiological data for identified cell types using tools such as calcium imaging (Chartier et al., 2018) and CRISPR-Cas9 (Bezares-Calderón et al., 2018). Importantly, the experimental techniques used for data collection (electron microscopy, *in situ* hybridization, and single-cell RNA sequencing) can be readily applied to non-canonical laboratory species. The comprehensive integration of whole-body connectomics and transcriptomics will open the door to the *in toto* comparison of cell types and neural circuits within and across organisms at the genetic and ultrastructure level, bringing us closer toward the understanding of the physiology, development, and evolution of living systems.

### Limitations of the study

Our success capitalizes on two main features of the *Platynereis* three-segmented young worm that may not apply to other model species. The small size of 6-dpf *Platynereis* allows acquiring an entire individual in one SBEM dataset (Titze et al., 2018), which is not yet possible for an equally differentiated stage of an insect or a vertebrate. Advances in EM will allow acquiring larger samples and expanding the range of accessible species. Also, the high stereotypy of *Platynereis* development is key to multimodal image registration at cellular resolution (Vergara et al., 2017) but may not apply to animals with more regulative development and random or mosaic cell arrangements (as in cnidarians or vertebrates). However, considerable degrees of stereotypy are present in other systems, such as nematodes (Schafer, 2016), *Drosophila* (Jenett et al., 2012), *Aplysia* (Katz and Quinlan, 2019), the chordate *Ciona* (Satoh, 1999), and early neurons of zebrafish (Metcalfe and Westerfield, 1990). Even in cases where cellular-resolution alignment cannot be obtained, we expect that multimodal registration will be possible at the level of genetically homogeneous cell types or tissues.

## STAR★Methods

### Key resources table

**Table:** 

REAGENT or RESOURCE	SOURCE	IDENTIFIER
**Antibodies**		

H3K36me3	Abcam	ab194677

**Critical commercial assays**		

RNeasy micro kit	QIAGEN columns	Cat. No. / ID: 74004
Click-iT EdU kit	Invitrogen	Catalog number: C10340

**Deposited data**		

The Electron Microscopy data and EM based segmentation	This paper	http://www.ebi.ac.uk/pdbe/emdb/empiar Accession id 10365
Gene expression maps, prospr segmentations, and registration files	This paper	https://www.ebi.ac.uk/biostudies Accession id S-BIAD14
The tabular data derived from images and segmentations	This paper	https://github.com/mobie/platybrowser-datasets/tree/master/data/1.0.1/tables

**Experimental models: Organisms/strains**		

6 dpf *Platynereis dumerilii*	EMBL Heidelberg	N/A

**Software and algorithms**		

SBEMImage	Titze et al., 2018	https://github.com/SBEMimage/SBEMimage
Virtual cell generation	This paper	https://github.com/mobie/prospr-vc-generation
EM segmentation, morphological, and genetic analysis of segmented cells	This paper	https://github.com/mobie/platybrowser-datasets
Intensity Correction	This paper	https://github.com/mobie/platybrowser-datasets/tree/master/misc/intensity_correction
SoftWoRx 6.2	GE Healthcare	N/A
Chromagnon image registration software	Matsuda et al., 2018	https://github.com/macronucleus/Chromagnon
SIMcheck	Ball et al., 2015	https://github.com/MicronOxford/SIMcheck
Mutex Watershed	Wolf et al., 2018	https://github.com/hci-unihd/mutex-watershed
Multicut solver	Pape et al., 2019	https://github.com/constantinpape/cluster_tools
Paintera	Hanslovsky et al., 2020	https://github.com/saalfeldlab/paintera
KNOSSOS	N/A	https://github.com/knossos-project/knossos
PyKNOSSOS	Wanner et al., 2016b	https://github.com/adwanner/PyKNOSSOS
TrackEM	Cardona et al., 2012	https://imagej.net/plugins/trakem2
elastix	Klein et al., 2010; Shamonin et al., 2014	https://github.com/SuperElastix/elastix
ElastixWrapper	Tischer, 2019	https://github.com/embl-cba/elastixWrapper
ImageJ	Schindelin et al., 2012	https://imagej.nih.gov/ij/
Ilastik	Berg et al., 2019	https://www.ilastik.org/
Python Louvain	N/A	https://github.com/taynaud/python-louvain
UMAP	McInnes et al., 2018	https://umap-learn.readthedocs.io/en/latest/
scikit-image	van der Walt et al., 2014	https://scikit-image.org/
scikit-learn	Pedregosa et al., 2012	https://scikit-learn.org
vigra	N/A	http://ukoethe.github.io/vigra/
mahotas	Coelho, 2012	https://mahotas.readthedocs.io/en/latest/
networkx	Hagberg et al., 2008	https://networkx.org/
pandas	McKinney, 2010	https://pandas.pydata.org/
scipy	Virtanen et al., 2020	https://www.scipy.org/
numpy	van der Walt et al., 2011	https://numpy.org/
snakemake	Köster and Rahmann, 2012	https://snakemake.readthedocs.io/en/stable/
tidyverse	Wickham et al., 2017	https://www.tidyverse.org/
rgl	CRAN	https://cran.r-project.org/web/packages/rgl/index.html
vegan	CRAN	https://cran.r-project.org/web/packages/vegan/vegan.pdf
MoBIE	This paper	https://github.com/mobie/mobie#mobie

### Resource availability

#### Lead contact

Further information and requests for resources should be directed and will be fulfilled by the lead contact, Detlev Arendt (arendt@embl.de).

#### Materials availability

Plasmids to generate *in situ* hybridization probes are available upon requests, no MTA required.

### Experimental model and subject details

*Platynereis dumerilii* larvae were obtained from an established culture at EMBL Heidelberg. Animals were kept and raised in natural seawater, at a constant temperature of 18°C, and under a 16 - 8 hours light - dark cycle. The study used animals at 6 days post fertilization.

### Method details

#### Sample fixation, preparation, and imaging

6 dpf *Platynereis dumerilii* were anaesthetised using 7% MgCl_2_ in seawater (1:1 ratio). *Platynereis* worms *were* fixed in a solution of 2% formaldehyde and 2.5% glutaraldehyde in 0.1 M sodium cacodylate buffer for 4 days at 4°C.

Fixed samples were prepared for SBEM following an adapted form of the NCMIR protocol (Deerinck et al., 2010) aided by microwave application (Pelco Biowave). Samples were postfixed with 2% osmium tetroxide in 1.5% potassium ferrocyanide (14 min of 2 min on/off cycles, 150 W, with vacuum) followed by rinsing with H_2_O. The rinse protocol, used throughout processing, involved one initial exchange of H_2_O on the bench and twice aided by the microwave (40 s, 80 W). The samples were then incubated in 1% aqueous solution of thiocarbohydrazide (14 min of 2 min on/off cycles, 150 W, with vacuum with the cold spot set to 40°C), followed by rinsing with H_2_O. This was followed by a second step of osmium tetroxide, this time in a 2% aqueous solution (14 min of 2 min on/off cycles, 150 W, with vacuum) and another rinse step. Samples were then incubated in 1% uranyl acetate (aqueous) overnight at 4°C. The following day the samples were rinsed and the final step of staining was performed. Samples were transferred to Walton’s lead aspartate solution for 14 min in the microwave (2 min on/off cycles, 150 W, vacuum, 50°C). Samples were again rinsed with H_2_O and then dehydrated with increasing concentrations of ethanol (20%, 50%, 70%, 90%, 3 × 100%). Samples were then infiltrated with durcupan resin through increasing percentages of resin with ethanol (25%, 50%, 75%, 3 × 100%). Samples were first prepared using the minimal resin method (Schieber et al., 2017) and then embedded in silver epoxy resin (Wanner et al., 2016a).

Samples were mounted onto aluminum pins with 2-part silver epoxy. Images were acquired with a ZEISS Merlin SEM at 1.8 keV landing energy, 270 pA beam current, and 0.8 μs dwell time. ∼2.5 TB comprising 11,416 slices with > 200,000 image tiles were acquired at 10 × 10 nm^2^ pixel size and 25 nm cutting thickness. During the seven-week-long acquisition we used the open-source acquisition software SBEMimage (https://github.com/SBEMimage/SBEMimage) (Titze et al., 2018). For image registration, translational offsets between neighboring image tiles were calculated using a custom optimized normalized cross-correlation procedure (Wanner et al., 2016a). Subsequently, pairwise relative offsets between neighboring tiles were used to optimize the tile positions in a global total least square displacement sense. Before in-plane stitching, the histograms of neighboring image tiles were matched in order to adjust and homogenize the contrast and brightness. A subset of about 10% of the sections showed nonlinear distortion artifacts due to sample charging. For these sections, a non-distorted neighboring section was manually chosen as a reference for distortion correction using the ImageJ plugin bunwarpJ (Arganda-Carreras et al., 2006).

Intensities of the fully stitched z-slices were then matched to remove intensity and contrast jumps in the z axis. This was done by calculating the 5% (L) and 95% (U) quantiles of intensity for each slice within the *Platynereis* (excluding the resin and silver embedding) from a downsampled version of the raw data (pixel size of 0.32 × 0.32 × 0.025 μm^3^). The 95% quantile U was matched between slices to adjust for shifts in absolute intensity, while the quantile range U - L was matched between slices to adjust for shifts in contrast. For each slice, these adjustments can be written as a linear transformation of the intensities: x_c_ = ((U-L)_ref_/(U-L)) (x–U) + U_ref_ where x_c_ are the corrected intensities of the slice, x are the raw intensities of the slice and ‘ref’ refers to the reference which was taken as the median of all slices. Using the median intensity of the resin around the *Platynereis* as a reference (as this should be fairly constant throughout the dataset), we noticed that this technique performed less well at the start and end of the dataset, where only the tips of the *Platynereis* remain. To correct for this, we chose a minimum z cutoff of slice 800 and a maximum z cutoff of slice 9800 – values beyond these cutoffs were corrected by the median of the correction factors of the 100 slices next to each cutoff (i.e., the calculated values are extrapolated for the very tips of the dataset).

Scripts for the intensity correction in *z* can be found on github: https://github.com/mobie/platybrowser-datasets/tree/master/misc/intensity_correction

#### Immunofluorescence labeling and 3D-SIM

H3K36me3 immunolabelling was performed on 4% PFA-fixed 6 dpf *Platynereis* specimens using a 1:200 concentration of the Abcam antibody ab194677. Super-resolution 3D structured illumination microscopy (SIM) of H3K36me3 immunolabelled and 4’, 6-diamidino-2-phenylindole (DAPI) stained specimens was performed on a DeltaVision OMX V3 Blaze system (GE Healthcare) equipped with sCMOS cameras (PCO), and 405, 488 and 593nm lasers, using a 60x NA 1.42 PlanApo oil immersion objective lens (Olympus). Specimens were mounted on a microscope slide with Vectashield and then covered with a No.1.5H (170 μm ± 5 μm tol.) coverslip (Marienfeld Superior). To adapt for spherical aberration when imaging in extended depth (10-20 μm) immersion oil with a refractive index (RI) of 1.518 was used. Raw data was acquired with a z-distance of 125 nm and with 15 raw images per plane (5 phases, 3 angles) and reconstructed with SoftWoRx 6.2 (GE Healthcare) using channel-specifically measured optical transfer functions (OTFs) generated from 100 nm diameter FluoSphere beads (ThermoFisher) recorded with 1.514 RI oil, respectively, and Wiener filter setting 0.0040 (Demmerle et al., 2017). Color channel alignment was performed using Chromagnon image registration software (Matsuda et al., 2018) using biological 3D calibration slides of simultaneously multicolor detected EdU pulse labeled mammalian cells. 3D-SIM raw and reconstructed data quality was assessed with SIMcheck (Ball et al., 2015). The full-scale 32-bit reconstructed data was thresholded for each channel to the stack modal gray value (representing the center of the background intensity level) and converted to 16-bit composite tif-stacks using the ‘threshold & 16-bit conversion’ utility of SIMcheck.

#### Segmentation Methods

In total, we provide segmentations of all cells, all nuclei, the cuticle and selected tissues and body parts as well as nuclear chromatin. For simpler segmentation tasks (tissues and regions of the animal: coelomic cavity, glands, gut, secretory cells and yolk) where the region boundary is very pronounced we use the carving workflow of ilastik (Berg et al., 2019) on downsampled data (80 × 80 × 100 nm^3^). Muscles and neuropil tissue were segmented at the same resolution, using a CNN for semantic segmentation. Similarly, to segment nuclear chromatin we use ilastik pixel classification, limiting it to the pre-segmented nuclei regions. For ilastik training the nuclei of 50 cells with diverse nuclear morphology were interactively annotated as “heterochromatin + nucleolus” or “euchromatin” classes, on data downsampled to 20 × 20 × 25 nm^3^ voxel size.

For the more complex tasks of cell and nuclei segmentation as well as for the segmentation of the cilia and cuticle, we extended the state-of-the-art EM segmentation methods originally developed for neural tissue blocks. In essence, the pipeline consists of a membrane detection step performed by a 3D U-net and a graph agglomeration step performed either by the Lifted Multicut or the Mutex Watershed algorithms.

In detail, we start from the segmentation of the nuclei. The groundtruth annotations for CNN training were provided by ariadne.ai (12 blocks of 400 × 400 × 120 pixels each) and additionally curated. A 3D U-Net (Çiçek et al., 2016) was trained to predict short- and long-range pixel affinities as described in Lee et al. (2017) and to predict for each pixel whether it belongs to a nucleus. The predictions were processed by the Mutex Watershed algorithm (Wolf et al., 2018), blockwise in blocks of 512 × 512 × 64 pixels. We chose Mutex Watershed over the more common superpixel-based Multicut agglomeration as we observed Multicut frequently merges individual nuclei which touch across a very small portion of their boundary (short-circuiting of the multicut constraints). The individual block segmentations were then stitched together using Multicut-based agglomeration with edge weights derived from pixel affinites as described in Pape et al. (2017). All computations were done on raw data downscaled to the resolution of 80 × 80 × 100 nm^3^.

Cilia and cuticle segmentations were performed using the same method as for the nuclei. The cilia segmentation was performed at full resolution (10 × 10 × 25 nm^3^ voxel size) but only applied to the segmented nephridia cells, using 3 blocks of annotated training data consisting of a total of 171 megavoxels. The cuticle segmentation was performed for data downscaled to 40 × 40 × 50 nm^3^ using 5 training blocks consisting of a total of 495 megavoxels.

Cell segmentation was also started from membrane detection. The groundtruth annotations were provided by ariadne.ai, consisting of 8 blocks of 628 × 628 × 130 pixels that were additionally curated and extended by one additional block of size 1280 × 1280 × 120 pixels to include more biological variability. A 3D U-net was trained to predict short- and long-range pixel affinities. In addition, we insert the edges of tissue and region segmentations (see above) into the affinity predictions, in order to avoid missing boundary signal due to the very different appearance of some region/tissue boundaries. These predictions were then used to break the volume into superpixels by the blockwise distance transform-based watershed algorithm (Beier et al., 2017). The superpixels were used to construct a region adjacency graph and to solve the segmentation problem as a graph partitioning with Lifted Multicut (Horňáková et al., 2017). Unlike (Beier et al., 2017) and other connectomics pipelines (Funke et al., 2019; Januszewski et al., 2018; Lee et al., 2017), we additionally exploit the nuclei segmentation to enforce separation of cells containing different nuclei. To that end we introduce lifted edges between superpixels which belong to the segmented nuclei, attractive for the superpixels of the same nucleus and repulsive for the superpixels of different nuclei. Lifted edges are introduced up to a graph distance of 4 and the attractive / repulsive edge weight is set to the maximum / minimum of the local edge weights. This approach adapts the common boundary based approach used for neuron segmentation to the larger variety of appearance found in the cellular segmentation task for the complete animal. The overall lifted multicut problem was solved by the hierarchical solver introduced in Pape et al. (2019). Since the nuclei repulsion is only included up to a certain graph distance, there are still objects in the resulting cell segmentation that contain more than one nucleus segment. We find these in post-processing and separate them individually by running a graph watershed seeded from the nodes mapped to the nuclei. Cell segmentation was performed on the raw data downscaled to a voxel size of 20 × 20 × 25 nm^3^; the runtime for the whole volume measured 10 hours on 6 GPUs for the neural network prediction and 20 hours on a CPU computer cluster for the agglomeration part. Several ganglia in the animal’s head were identified by manually selecting the corresponding segmented cells.

We leverage prior information from nuclei and tissue segmentation to mitigate segmentation errors arising from ruptured cellular membranes and diverse appearance of cell boundaries. While these issues could be mitigated by providing additional training data, this process is very laborious for 3D segmentation. Instead, we prefered to rely on the nuclei segmentation - a much simpler problem which our algorithm solves to 99.0% accuracy - and tissue segmentation, which can be achieved at lower resolution. Our use of nuclei as prior knowledge assumes that every cell should contain only one nucleus, a constraint that is expected to be true for all cells (including muscles) at this developmental stage.

The final proof-reading was performed in a semi-automated manner. First, we compute a morphology-based score for all cells that roughly matches the likelihood of a cell being a false merge. Then, we iterate through the top 1000 cells based on their rank by this score and correct all cells that contain a false merge by running graph watershed from user-generated seeds. This resolving step had to be applied to 154 falsely merged cells. In addition, we use Paintera (Hanslovsky et al., 2020) to perform some more fine grained proof-reading. Note that the segmentation currently provided in the PlatyBrowser has not been proof-read down to the pixel level of every cell. We corrected the errors we found by the approach above and additionally polished the segmentation of the regions used in the analysis presented here: nephridia, adult eyes, symmetric cells used for the gene assignment validation. For reference, finalising the segmentation of the nephridia (14 cells with far-reaching cilia) from the fully automatic pipeline results took approximately 2 hours with Paintera.

In addition, semantic segmentations based on a CNN for the muscles and the neuropil were provided by ariadne.ai.

We provide the weights for the networks used to segment cell membranes, cilia, cuticle and nuclei on zenodo https://zenodo.org/record/3675288 as well as the corresponding training data https://zenodo.org/record/3675220. The ilastik project and training data for the chromatin segmentation is also available at https://zenodo.org/record/3676534. The ilastik projects for carving the animal outline and regions/tissue are available at https://zenodo.org/record/3678793.

The scripts to run the segmentation methods are available at https://github.com/mobie/platybrowser-datasets/tree/master/segmentation.

#### Segmentation Validation

The validation of the segmentation was based on manual annotations for cell centers and nuclei on 8 slices (4 transversal, 4 horizontal) from 8 domain experts, each slice annotated at least twice without access to the automatic segmentation results. The missing detections in the expert annotations were then additionally corrected by comparison with the automatic segmentation. We provide the validation data at https://zenodo.org/record/3690727.

In total we observe, for the cell segmentation task, 6.30% false split errors and 3.23% false merge errors, based on 4806 annotations. For the nucleus segmentation task, we found 0.49% false positive detections; 0.55% false negative detections based on 2888 annotations. Figure S1G gives an overview of the expert annotations and 2 examples of false merge and false split errors each.

#### Groundtruth annotation

The groundtruth annotations for training the automated segmentation pipelines were generated and curated by human expert annotators in the open-source 3D annotation software KNOSSOS (https://knossos.app/, https://github.com/knossos-project/knossos).

#### Neuron tracing

The morphology of each neuron was traced using the open-source tracing software PyKNOSSOS (https://github.com/adwanner/PyKNOSSOS) (Wanner et al., 2016b). Starting from seed points in somata, human expert annotators manually reconstructed the skeleton of the neurons by following their neurites within the 3d EM volume. Neurite branch points were manually identified and tagged. In an iterative procedure, the different neurites originating from a given branch point were reconstructed. In total 384 neurons have been reconstructed manually with a total length of 36.94 mm, which took a total of 304.51 hours of manual tracing. All reconstructed neurons are available in the PlatyBrowser and the skeleton files can be downloaded from https://zenodo.org/record/4660134.

#### Expansion of the 6dpf ProSPr atlas

To obtain comprehensive expression information for all cells in the body at 6 dpf, we introduced new genes into the 6dpf ProSPr resource focusing on areas with poor expression coverage in previous versions of the atlas, such as the digestive system. For example, we selected 37 genes that we found highly enriched in a foregut cDNA library (see section below) and that proved to be differentially expressed in the foregut. We also introduced additional genes with preferential nervous system expression, with special focus on neural differentiation genes. As a result, the 6 dpf ProSPr gene expression atlas (referred to as ProSPr atlas) now contains information for 201 genes (including 78 transcription factors, 56 neural effector genes, and 53 other differentiation genes) and 4 EdU proliferation stainings (see below).

#### Foregut cDNA library

Around 40 maturing animals (juveniles) were isolated from the tubes and starved for three days. The epidermis around the foregut was removed to reveal the muscular pharynx and the anterior part of the organ. The tissue was immediately fixed in liquid nitrogen. Tissue was then ground in Trizol and a Trizol/Chloroform extraction was performed. RNA was purified with the RNeasy kit (QIAGEN columns). Illumina library was prepared by the EMBL GeneCore facility. cDNA for individual gene cloning was generated with SuperScript III kit (Invitrogen).

#### *In situ* hybridization and registration

Animal breeding, animal fixations, and whole-mount *in situ* hybridizations were performed as described previously (Tessmar-Raible et al., 2005). Light microscopy sample imaging and gene expression maps were generated following the ProSPr protocol as described in Vergara et al. (2017). Animal body parts (head, cryptic segment, ventral nerve cord, foregut, lateral ectoderm and pygidium) were manually segmented in TrackEM (Cardona et al., 2012) using the ProSPr DAPI reference.

#### EdU proliferation assays and tracing analysis

We incorporated EdU (5-ethynyl-2′-deoxyuridine) at 200 mM directly in sea water during specific developmental periods (42 hours to 48 hours; 3 days to 4 days; 4 days to 5 days; 5 days to 6 days) in order to label the nuclei of those cells undergoing mitosis within a certain period. Fluorescent labeling was done using the Click-iT™ EdU kit following the manufacturer’s instructions (Invitrogen).

We categorized EdU positive cells from the period 4 to 6 days old as late dividing and should represent immature neurons and/or progenitor cells. We hypothesize that this should be reflected in a lack of long neuronal processes. To test this, we randomly selected 10 EdU positive cells and 10 EdU negative cells and traced their morphology. Only 1 out of 10 of the EdU positive cells showed a projection to the central neuropil, contrasting with 9 out of 10 of the EdU negative cells, confirming that these cells are still developing and constitute proliferation domains. This correlation adds additional support to the accuracy of the registration between the ProSPr atlas and the electron-microscopy dataset.

#### Registration of ProSPr to EM

As the typical variation between individuals in the position of individual cells is less than one cell diameter (< 4.7 μm) (Vergara et al., 2017), we reasoned that registration should be possible at close to cellular resolution. To avoid compromising the appearance of ultrastructural morphology, we computed a transformation of the lower resolution ProSPr atlas onto the higher resolution SBEM volume. We used the software package elastix (Klein et al., 2010; Shamonin et al., 2014) to compute a multi-step registration of the average DAPI signal (representing nuclei of 153 specimens from the ProSPr atlas) onto the binary mask of the segmented nuclei of the EM individual (Figure S3C). Historically, elastix is mainly used by the medical image analysis community. To increase the usability of elastix for the biological community we developed the Fiji (Schindelin et al., 2012) plugin ElastixWrapper (Tischer, 2019), which we used for the registration. As an input for the registration we prepared three image files at an isotropic voxel spacing of 0.55 μm: DAPI: a greyscale image of the average DAPI signal of ProSPr (Vergara et al., 2017); EM-Nuclei: a binary image containing the segmented EM nuclei; and EM-Mask: a binary mask covering the image region containing information relevant for the registration optimization (Figure S3). To prepare the EM-Nuclei image we removed 42 nuclei in the gut region from the nuclei segmentation binary mask as these nuclei do not locate to stereotypical positions and were not reflected in the ProSPr DAPI signal, and therefore they did not properly guide the registration. Next, we downsampled the binary mask of the remaining 11,456 segmented nuclei to the 0.55μm voxel size of the ProSPr data; note that due to the bi-linear downsampling algorithm the voxel values in the EM-Nuclei image are not strictly binary, but show some shades of gray (Figure S3B). To prepare the EM-Mask image we isotropically dilated the EM-Nuclei image by 12 pixels, corresponding to 6.6 μm. The EM-Mask serves to restrict the optimization algorithm of elastix to relevant regions of the image. The usage of such a mask in elastix is optional, though we found it critical for our image data because it contains a lot of empty space due to the oblique orientation of the EM image within the 3D voxel space. Using these images we sequentially ran elastix with transformation models of increasing deformability: Similarity: a rigid transformation, allowing for rotation, translation, and a uniform scaling factor; BSpline100: a locally deformable BSpline transformation (Rueckert et al., 1999) with a 3-D voxel grid spacing of 100 pixels (55 μm); BSpline30: BSpline transformation with a grid spacing of 30 pixels (16.5 μm); BSpline10: BSpline transformation with a grid spacing of 10 pixels (5.5 μm). The registrations build on each other, e.g., the BSpline100 transformation takes the Similarity transformation as a starting point. Importantly, since the two datasets are initially very misaligned (Figures S3A and S3B) using the automated registration algorithms of elastix directly did not work for us. The reason is that elastix uses local search algorithms for improving the transformation parameters and thus typically needs a decent starting condition. To produce such a starting condition, we used the Fiji plugin TransformJ Rotate (https://imagej.net/libs/imagescience) to manually determine angles that roughly align the two datasets in 3-D. We then converted these angles into an elastix transformation file (Manual-Rotation). Using the Manual-Rotation transformation as a starting point, we could then use elastix to sequentially improve the registration. After each step in the registration sequence we visually compared the transformed DAPI image with the EM-Nuclei image to qualitatively assess whether the registration improved (e.g., using overlays as shown in Figure 4A in the main text). To further assess registration quality, we manually identified 43 corresponding landmarks between the EM and ProSPr datasets, covering all relevant regions of the specimen (Figure S3D). These landmarks were selected based on unequivocal positions in both datasets using the nuclei, muscle and neuropil signals. Using these landmark pairs we could measure the registration quality at each step by transforming the manually assigned landmark coordinates in EM space into ProSPr space and then comparing these computed coordinates with the manually assigned coordinates. Measuring the pairwise distances of the 43 coordinate pairs for the different registration steps we obtained the following results (mean, median): Manual-Rotation: 63.57 μm, 58.79 μm; Similarity: 11.90 μm, 10.09 μm; BSpline100: 5.29 μm, 4.48 μm; BSpline30: 4.23 μm, 3.53 μm; BSpline10: 3.47 μm, 2.99 μm. The fact that the mean and median are similar shows that there are no severe outliers and thus the registration quality is consistent across all parts of the animal (Figures S3D and S3E). In addition, the pairwise distances decrease with each registration, showing that the elastix algorithm indeed converged to a better registration. We did not attempt to improve the registration beyond the current measured accuracy of ∼3 μm, because at 6 dpf the typical variation in the position of individual cells in *platynereis* is ∼4.7 μm (Vergara et al., 2017).

Scripts and transformation files used for the registration can be found on github: https://github.com/mobie/platybrowser-datasets/blob/master/registration/0.6.3

#### Morphology clustering

Various morphological, intensity and texture features were calculated from the cell, nucleus and chromatin segmentations (full description of features in Table S1). Cells (and their associated nuclei and chromatin) were filtered to remove those most likely to be affected by segmentation errors – cells were removed that had no assigned nucleus, were in certain regions (yolk, neuropil, cuticle, cavities), or outside a reasonable size range. This resulted in 11348 remaining cells. All features were calculated on data downsampled to 80 × 80 × 100 nm^3^ resolution.

For downstream analysis, any cells (and associated nuclei and chromatin) that were within the region for extrapolated intensity correction (see Sample fixation, preparation and imaging methods section) were also discarded (leaving a total of 10344 cells). This was to avoid any possible artifacts in features that rely on the raw intensity data.

This resulted in a table of 140 features by 10344 cells. All features were standardized by centring to a mean of 0 and scaling to unit variance. A K-nearest neighbor graph was then constructed (k = 10) using Euclidean distance between the feature vectors. Community detection (using the Louvain method (Blondel et al., 2008)) was performed with a resolution parameter of 1.2, resulting in 11 clusters. Clusters were visualized on a UMAP (McInnes et al., 2018) (Uniform Manifold Approximation and Projection for Dimension Reduction) using the following parameters: n_neighbors = 10 and min_dist = 0.1.

All the morphological clustering analysis was performed in Python with scikit-image (van der Walt et al., 2014), vigra (http://ukoethe.github.io/vigra/), scipy (Virtanen et al., 2020), mahotas (Coelho, 2012), scikit-learn (Pedregosa et al., 2012), networkx (Hagberg et al., 2008), python-louvain (https://github.com/taynaud/python-louvain), umap-learn (McInnes et al., 2018), pandas (McKinney, 2010), numpy (van der Walt et al., 2011) and snakemake (Köster and Rahmann, 2012). All code is freely available in the github repository (the clustering code as a snakemake workflow). The snakemake workflow also contains code for various other analyses e.g., clustering of different subsets of morphological features, plotting of *Platynereis* regions on the UMAP, plotting gene expression on the UMAP, or construction of various heatmaps.

The script for calculation of morphological features is available here: https://github.com/mobie/platybrowser-datasets/blob/master/mmpb/extension/attributes/morphology_impl.py and the Snakemake workflow for clustering analysis is available here: https://github.com/mobie/platybrowser-datasets/tree/master/analysis/morphology_clustering. The final clustering is available here: https://github.com/mobie/platybrowser-datasets/blob/master/data/1.0.1/tables/sbem-6dpf-1-whole-segmented-cells/morphology_clusters.csv and the final UMAP here: https://github.com/mobie/platybrowser-datasets/blob/master/data/1.0.1/tables/sbem-6dpf-1-whole-segmented-cells/morphology_umap.csv.

#### Cell type annotation

In order to be able to investigate morphological and genetic differences between cell types, segmented cells were annotated as follows. First, seven morphological distinct cell categories were defined: neurons, dark cells (cells with very dark, uniform nuclei and cytoplasm), epithelial, muscle, digestive midgut cells, secretory (a broad category of any cells with prominent endoplasmic reticulum, Golgi and secretory vesicles / inclusions) and ciliated cells (multiciliated cells on the surface) (Figure S2A). Second, all the 11402 segmented cells were split into mini-blocks of consecutive 10 cells. In order to ensure uniform sampling across the animal, these mini-blocks were randomly shuffled and merged together into bigger blocks, consisting of 100 cells each. Random 10 of the resulting 115 blocks were manually annotated using the annotation functionality of the MoBIE viewer. The annotators could assign one of the abovementioned cell classes or the labels ‘unsure’ or ‘error’. As a result, 65% of the cells were annotated as neurons, 16% as epithelial cells, 11% as muscle cells, 3.5% as dark cells, 2.38% as midgut cells, 1.80% as secretory cells and 0.35% as ciliated cells. Afterward, more manual annotation was done specifically targeting the underrepresented cell classes: ciliated, secretory, midgut and dark cells. This resulted in 571 neuron, 141 epithelial cells, 53 muscle cells, 41 dark cells, 25 ciliated cells, 51 secretory cells and 55 midgut cells. The annotation results are available here: https://github.com/mobie/platybrowser-datasets/blob/master/data/1.0.1/tables/sbem-6dpf-1-whole-segmented-cells/default.csv (column ‘cell_type’).

#### Bilateral pair analysis

To assess how different subsets of morphological features (Table S1) perform on finding bilateral pairs of cells in the dataset, we established a set of xyz criteria for being bilateral based on the position of the cell’s nuclei. As the *Platynereis* is bent in the EM image volume, we cannot directly use the xyz position of the nuclei to determine if they are bilateral or not. Instead, we first calculated a midline surface for the *Platynereis* by fitting a second order polynomial of two variables to a set of manually chosen points that lie on the midline. The absolute distance from this surface could then be calculated for all nuclei. While this accounts for the distance from the midline, it doesn’t assess if nuclei have a similar position along the anterior-posterior (AP) or dorsal-ventral (DV) axis of the *Platynereis*. To account for this, we transformed the xyz position of all nuclei from the EM space back to the original ProSPr space via the program elastix (Klein et al., 2010; Shamonin et al., 2014) (same transformation parameters as calculated for the registration – see Registration of ProSPr to EM methods section). As the AP and DV axes are nicely aligned to y and z in the ProSPr space, we can use these coordinates as estimates for AP and DV position. The full criteria are then: similar absolute distance from midline; on opposite sides of the midline; similar y position in ProSPr space; similar z position in ProSPr space.

To assess a reasonable range to consider ‘similar’, these statistics were calculated for a set of manually curated bilateral pairs of cells (202 pairs total). The acceptable difference was then set to the mean over these pairs plus two standard deviations.

Given these criteria to assess if cells are bilateral, we then wished to calculate the distance in morphology space that must be traveled from one cell to find its potential bilateral partner. This was calculated first by taking a certain subset of the morphology criteria (calculated as in the Morphology clustering methods section) and standardizing them – by centring each feature’s mean to 0 and scaling to unit variance. For each cell, a ranking was then formed (based on Euclidean distance in morphology space) of every other cell in the dataset - from its very closest neighbor, to its most distant neighbor. Then, using the xyz criteria above, the closest neighbor was found in this ranking that was a potential bilateral partner.

The subsets used in Figure 3H were: ‘all’ (all morphology features), ‘cell’ (only cell features), ‘nucleus’ (all nucleus and chromatin features), and ‘chromatin’ (only intensity and texture features of chromatin).

To compare to random assignment of bilateral pairs, the ranking of cells in morphology space was randomly shuffled, and the same analysis performed. This was repeated 100 times, and the mean of all trials taken for the final randomized results shown in Figure 3H.

Scripts for this analysis are available here: https://github.com/mobie/platybrowser-datasets/tree/master/analysis/bilateral_pairs , and the midline calculation is available here: https://github.com/mobie/platybrowser-datasets/tree/master/analysis/midline. The table of manually curated pairs is available here: https://github.com/mobie/platybrowser-datasets/blob/master/data/1.0.1/tables/sbem-6dpf-1-whole-segmented-cells/symmetric_cells.csv. The midline fit and final graphs were calculated in R, making use of the tidyverse (Wickham et al., 2017) and rgl (https://cran.r-project.org/web/packages/rgl/index.html) packages. All other analysis was in Python with pandas (McKinney, 2010), numpy (van der Walt et al., 2011), scikit-learn (Pedregosa et al., 2012) and scipy (Virtanen et al., 2020).

#### Gene expression clustering

Overlap assignment was used to assign a vector of gene overlap values to each segmented cell. In brief, the fraction of the volume of each segmented cell overlapping with the registered volume of every gene was calculated. This resulted in a vector of length 201 (equal to the number of genes) with values ranging from 0 (no overlap) to 1 (complete overlap).

Segmented cells were then filtered to remove those most likely to be affected by segmentation errors – cells were removed when they had no assigned nucleus, when they were in certain regions (yolk, neuropil, cuticle, cavities), or when they were outside a reasonable size range. In addition, any cells that expressed no genes were discarded. This resulted in 11,366 remaining cells.

A K-nearest neighbor graph was then constructed (k = 20) using Euclidean distance between the gene overlap vectors. Community detection (using the Louvain method (Blondel et al., 2008)) was performed with a resolution parameter of 1.2, resulting in 15 clusters.

Clusters were visualized on a UMAP (McInnes et al., 2018) (Uniform Manifold Approximation and Projection for Dimension Reduction) using the following parameters: n_neighbors = 20 and min_dist = 0.1. All the gene expression clustering analysis was performed in Python with scikit-learn (Pedregosa et al., 2012), networkx (Hagberg et al., 2008), python-louvain (https://github.com/taynaud/python-louvain), umap-learn (McInnes et al., 2018), pandas (McKinney, 2010), numpy (van der Walt et al., 2011) and snakemake (Köster and Rahmann, 2012).

This section used version 1.0.0 of the data. The code is freely available as a snakemake workflow: https://github.com/mobie/platybrowser-datasets/tree/master/analysis/gene_clustering. The snakemake workflow also contains code for various other analyses e.g., clustering of different kinds of gene expression (binarised by a threshold, assignment by virtual cells etc), plotting of *Platynereis* regions on the UMAP, plotting morphology statistics on the UMAP, or construction of various heatmaps. The final clustering is available here: https://github.com/mobie/platybrowser-datasets/blob/master/data/0.6.5/tables/sbem-6dpf-1-whole-segmented-cells/gene_clusters.csv and the final UMAP here: https://github.com/mobie/platybrowser-datasets/blob/master/data/1.0.0/tables/sbem-6dpf-1-whole-segmented-cells/gene_umap.csv.

#### Ganglia and gene specificity analysis

The detail in the EM volume and cell segmentation allowed us to classify and group the cells in the animal’s head in a complete and unbiased manner by using ultrastructural landmarks such as connective tissue and basal membranes (as compared to previous efforts recognizing brain anatomy mostly based on immunohistochemistry (Chartier et al., 2018; Tomer et al., 2010)).

Segmented head ganglia were displayed on the same UMAP as the gene expression data (see Gene expression clustering methods).

A specificity score was calculated for every gene cluster and individual gene for every ganglia. This specificity score is a combination of two measures: first, the fraction of expression confined to a given ganglion (A) and second, the fraction of that ganglion covered (B). In analogy to the F1 score, specificity is calculated as: 2AB / (A + B)

The calculation for individual genes relies on overlap assignment to segmented cells (see Gene expression clustering methods). A threshold of 0.5 was used to label a cell as expressing a particular gene.

The specificity calculations are part of the same workflow as described in the Gene expression clustering section: https://github.com/mobie/platybrowser-datasets/tree/master/analysis/gene_clustering , specific script here: https://github.com/mobie/platybrowser-datasets/blob/master/analysis/gene_clustering/scripts/ganglia_specificity.py , and also used version 1.0.0 of the data.

The MB ganglia genetic heatmap was generated by the following method. Cells are grouped into clusters (genetic territories) obtained via louvain community detection (as described in section ‘Morphology clustering’). Genes were sorted hierarchically and grouped into modules respecting the hierarchy of the tree (gene modules). For each module and genetic territory, we calculated the average expression overlap value, which we use to derive a specificity score as described above.

#### Generation of virtual cells

The generation of Virtual Cells (VCs) was done as in Vergara et al. (2017) with some modifications (see also main text). Analysis of ProSPr data and generation of VCs was done using the software R Bioconductor (Gentleman et al., 2004). The atlas was binned by a factor of 3 to generate SuperVoxels (SVs) - cubes of 27 isotropic voxels of 0.55 μm side (each SV represents a cube of 1.65 μm edge length and 4.5 μm^3^ volume). On average, and given the size of a nucleus in the EM dataset (67.32 ± 15.19 μm^3^), each cell is minimally covered at least by 12 SVs. Note that we used the nucleus size to estimate cell coverage as the big majority of cells are neurons (small cytoplasm), and supervoxels along cell boundaries will result in noisy signals. Next, we generated a matrix with the SV spatial location in xyz and the proportion of pixels within that SV positive for each gene. SVs with very low correlation with their neighbors were removed from the analysis as they represent noise. Due to memory constraints, the SVs were subdivided into different anatomical regions (e.g., head, ventral nerve cord, foregut, etc) for further analysis.

Next, SVs were grouped into Virtual Cells (VCs). This is done by a recursive process of hierarchical clustering to find groups of SVs based on their similarity in expression profile (Figure S6A). This process does not take into account the location of the SVs, but it is constrained by the final size of the group, allowing groups to be formed so that they represent between 1 and 6 cells, always optimizing for expression correlation. For hierarchical clustering we used the function ‘hclust’ with the method *‘complete’*. For distance calculation we used the method *‘jaccard’* from the *‘vegan’* package. For computing SVs correlation we used the basic R function ‘*cor*’ with the following parameters: *‘ use = “complete.obs,” method = “kendall” ’*. We found the majority of clusters comprising spatially confined groups of SVs with bilateral symmetry, which is a good indication of the high quality of the atlas.

In contrast to Vergara et al. (2017), VCs were automatically curated based on size and spatial distribution of their constituent SVs. In more detail, first all the VCs that contain less than 16 supervoxels were filtered out, since below this size they consistently showed low spatial correlation (probably representing cell boundaries). Next, VCs were split into spatially connected components (CCs), and the ones below size 5 supervoxels in size were filtered out (cell size values below 5 SVs do not properly represent a cell). All CCs comprised of 8 or more supervoxels were kept without further checks, and the ones in the range of [5,8) were checked for a symmetric partner - if there was another CC of the same VC symmetrically located on the other side of the animal, the CC was considered plausible and retained. The partner CC was considered symmetric if located within the radius of 4 supervoxels (one cell diameter) from the mirrored coordinates of the center of the given CC. After this curation step the VCs are assembled together again (so they are retained as bilateral cell types) and their expression profile is calculated.

The complete automated pipeline for generating Virtual Cells from the ProSPr gene expression maps can be found on GitHub: https://github.com/mobie/prospr-vc-generation.

#### Virtual cell assignment

In order to assign the gene expression patterns to the segmented cells the Virtual Cells (VCs) volume was registered onto the EM one. To compensate for biological variability and registration error for each cell we considered all the VCs found in the radius of 5 μm from the cell boundaries. The expression pattern of the cell calculated by gene overlap (Figures 7A and S6B) was compared to the expression pattern of every VC found. Finally, the segmented cell was assigned to the genetically closest VC in the vicinity (Figures 7B and S6C). Considering the fact that the available 201 genes were mostly targeting specific tissues, the animal can and does have areas without or with minimal gene coverage. That is why in case for a given cell either no VCs were found around, or an absence of any expression was genetically closer than any of the VC’s found, the cell was allowed not to be assigned any gene expression or, more precisely, to be assigned zero expression for all the genes.

Notably, although the spatial location of the assigned virtual cells (Figure S6D) is consistent with their spatial location in ProSPr space (Figure S6A), only in 39% of cases the segmented cell was actually assigned the spatially closest Virtual Cell. This shows that biological variability combined with the registration error limits the precision of cellular assignment based on expression overlap only.

In order to assess the assignment accuracy we used morphologically identifiable symmetric cell pairs that are supposed to have identical gene expression profiles. In total, 208 unambiguous symmetric pairs of segmented cells were marked in the EM volume, covering most tissues (Figures S6H and S6I). For each cell pair, we calculated the difference between genetic profiles assigned to the cells in the pair. This difference was compared for overlap versus virtual cell assignment (Figure S6G).

To compare this type of assignment to purely spatial assignment we also assigned the Virtual Cells by overlap: each segmented cell was assigned either the VC that overlapped its volume the most, or no expression in case no VC overlap was present. Such assignment gave a worse result in the symmetric cells test: the mean gene distance between symmetric cells was 3.62 (compared to 2.0 distance for assignment by genetic distance described above).

This analysis used version 1.0.0 of the data. Only segmented cells that contained nuclei were used for the analysis.

#### MoBIE and the PlatyBrowser

To make available the Platynereis atlas to the community we developed MoBIE (https://mobie.github.io). MoBIE is a free and open-source platform for multi-modal big image data exploration and sharing. It consists of an S3 object store (https://en.wikipedia.org/wiki/Amazon_S3) backend for cloud based image data sharing (Bogovic et al., 2020), GitHub based storage of tabular data and project metadata, and an easy to install Fiji (Schindelin et al., 2012) viewer for integrated browsing of the whole dataset.

The PlatyBrowser uses the MoBIE framework for the interactive browsing of our *Platynereis* atlas data. To this end, the MoBIE viewer fetches data from the https://github.com/mobie/platybrowser-datasets/ GitHub repository (see above section on Data Provision). To ensure consistent data access we implemented a git-based versioning system to accommodate further improvements of the registration or segmentations, as well as contributions of entirely new imaging modalities from the community. The repository contains different versions of the data; the most recent version at time of publication is 1.0.1 and the reported results are based on this version unless noted otherwise.

The MoBIE Fiji viewer is written in Java and thereby executable on all major platforms (Linux, Mac, Windows). The code is publicly available in a GitHub repository (https://github.com/mobie/mobie-viewer-fiji), which also contains the latest installation and usage instructions (https://github.com/mobie/mobie-viewer-fiji#installation). The graphical user interface of the MoBIE Fiji viewer is implemented using the Java Swing library (https://en.wikipedia.org/wiki/Swing_(Java)). The image data handling and visualization is based on imglib2 (Pietzsch et al., 2012), a java library for processing large N-dimensional image data. For image data visualization, the MoBIE viewer uses BigDataViewer (BDV) (Pietzsch et al., 2015). BDV enables arbitrary plane slicing of volumetric image data. This viewing modality is ideal for the exploration of our SBEM data, which is too dense for volume rendering. In addition, BDV supports simultaneous display of image data of different resolutions, thereby enabling the overlay of ProSPr (0.55 × 0.55 × 0.55 μm^3^) and SBEM (10 × 10 × 25 nm^3^) images (e.g., Figures 4B–4H).

Thanks to lazy-loading from a chunked pyramidal image file format (Pietzsch et al., 2015), everyone with an internet connection is able to browse the TB-sized atlas from a standard computer. The PlatyBrowser is thus an accessible and extendable resource for the investigation of *Platynereis dumerilii* ultra-structural morphology and gene expression.

In order to efficiently explore complex datasets, we designed a user interface for choosing from hundreds of image sources of different modalities as well as for adjusting their display settings (Figures 7D and 7G). The registration of the ProSPr atlas to the SBEM dataset permits to compute a (ProSPr based) gene expression profile at each location in the SBEM space. To enable interactive exploration of this information the PlatyBrowser provides the possibility to query gene expression profiles at each location, presented as both a ranked list and gene expression table (Figure 7G).

To explore segmentations, MoBIE provides adjustable lookup tables (Glasbey et al., 2007; Hanslovsky et al., 2020) to overlay each segment with a distinct color onto the image data. Much of this code was conceptualised and developed during a “Fiji hackathon” in Ostrava (Czech Republic, January 2019), and we are very grateful to Tobias Pietzsch, who, during this event, provided invaluable support with the software design. We use this functionality to visualize nuclei, tissue, and cell segmentations on top of the image data (Figures 7E and 7G). This makes it possible to visually observe whether distinct tissue regions correlate with specific ultrastructural morphology. Often, not only ultrastructural morphology but also the 3D shape of anatomical regions is indicative of biological function. We therefore added the possibility to render segmented cells in an interactive 3D viewer (Schmid et al., 2010) (Figure 7G).

The nuclei, cell, and tissue segmentations allow for the extraction of features such as cellular morphological descriptors or gene expression profiles (Figures 3 and 4). To interactively explore these measurements, MoBIE displays a table for each segmentation, where table rows correspond to segmented objects and table columns to feature values. Tables can be sorted according to any feature value and objects of special interest can be selected (highlighted) both in the viewer and in the table. Importantly, the coloring of objects can be configured to reflect any feature value, making it possible to overlay object-based measurements on the image data (see Figures 3B, 3G, 5C, and 5D).

In addition, we implemented a functionality to manually assign object annotations by making the tables editable. For example, we have used this feature to assign the cells in the head to ganglia. We also implemented a bookmark function that not only stores positions and views, but can also update the whole browser state, including segmentations, tables, and objects rendered in the 3D viewer. Bookmarks were used to provide interactive versions for many figure panels presented in this manuscript (see figure legends in the main text).

### Quantification and statistical analysis

Where appropriate, the quantitative analyses are described in the relevant sections of the Method details.

### Additional resources

In addition to the methods described above, we have generated documentation for the MoBIE Fiji plugin, which can be found at https://mobie.github.io.

## Data Availability

All data and code generated during this study are publicly available. The Electron microscopy data and EM based segmentation are archived and can be downloaded from EMPIAR (https://www.ebi.ac.uk/pdbe/emdb/empiar/) under the accession id 10365. Gene expression maps, prospr segmentations, and registration files are archived and can be downloaded from BioStudies (https://www.ebi.ac.uk/biostudies/) under the accession id S-BIAD14. In addition to EMPIAR and BioStudies we host all image data on an aws-s3 (http://aws.amazon.com/s3) compatible object store at EMBL Heidelberg using the open source implementation of MinIO (https://min.io). The data are stored in the n5 data format (Bogovic et al., 2020), which allows chunking and compression for efficient data access. It can be accessed on demand through the n5 s3 API (https://github.com/saalfeldlab/n5-aws-s3). The tabular data derived from images and segmentations are hosted on github: https://github.com/mobie/platybrowser-datasets/tree/master/data/1.0.1/tables. The repository https://github.com/mobie/platybrowser-datasets also contains the image metadata in the BigDataViewer xml file format, which serves as an entry point to start the PlatyBrowser for the data hosted on the s3 object store. In order to keep track of changes in the derived data, for example due to segmentation corrections, we use a versioning scheme inspired by https://semver.org/. Between each version only the data that has actually changed is updated, whereas unchanged data are referred to by links to older versions. The software package used for EM acquisition SBEMImage is available at https://github.com/SBEMimage/SBEMimage and described in the publication (Titze et al., 2018). The software for generating the virtual cells from ProSPr is available at https://github.com/mobie/prospr-vc-generation and under the https://doi.org/10.5281/zenodo.4899523. The software for EM segmentation and morphological as well as genetic analysis of segmented cells is available at https://github.com/mobie/platybrowser-datasets and under the https://doi.org/10.5281/zenodo.4899527. The MoBIE Fiji viewer plugin is available at https://github.com/mobie/mobie-viewer-fiji and under the https://doi.org/10.5281/zenodo.2602754.

## Supporting citations

The following references appear in the supplemental information: Haralick et al. (1973).
