# Supplementary material for: Whole-body integration of gene expression and single-cell morphology
Source: Cell. 2021 Sep 2;184(18):4819–4837.e22. doi: 10.1016/j.cell.2021.07.017 (PMC8445025; doi:10.1016/j.cell.2021.07.017)
Supplement: Document S1. Table S1 [file mmc1.pdf]

Cell, Volume *184*

Supplemental information

Whole-body integration of gene expression and single-cell morphology

Hernando M. Vergara, Constantin Pape, Kimberly I. Meechan, Valentyna Zinchenko, Christel Genoud, Adrian A. Wanner, Kevin Nzumbi Mutemi, Benjamin Titze, Rachel M. Templin, Paola Y. Bertucci, Oleg Simakov, Wiebke Dürichen, Pedro Machado, Emily L. Savage, Lothar Schermelleh, Yannick Schwab, Rainer W. Friedrich, Anna Kreshuk, Christian Tischer, and Detlev Arendt

Feature id	Segmentations	Description
Shape_volume_ in_microns	Cell, nucleus, H+N, euchromatin	Volume in μm^3
shape_extent	Cell, nucleus, H+N, euchromatin	Ratio of pixels in the object to pixels in the total bounding box
shape_equiv_diameter	Cell, nucleus, H+N, euchromatin	Equivalent diameter - the diameter of a sphere with the same volume as the object
shape_major_axis	Cell, nucleus, H+N, euchromatin	Length of the major axis of the fitted ellipsoid
shape_minor_axis	Cell, nucleus, H+N, euchromatin	Length of the minor axis of the fitted ellipsoid
shape_surface_area	Cell, nucleus, H+N, euchromatin	Surface area of object mesh (mesh calculated by the Lewiner marching cubes algorithm)
shape_sphericity	Cell, nucleus, H+N, euchromatin	Measure of how spherical an object is (0-1 scale, with 1 being a perfect sphere) calculated as $36\pi V^2/S^3$ where V is the volume of the object, and S is its surface area.
shape_max_radius	Cell, nucleus, H+N, euchromatin	Maximum distance from a pixel within the object to the outside (Euclidean distance)
intensity_mean	Cell (excluding nucleus), nucleus, H+N, euchromatin	Mean intensity of the segmented object
intensity_st_dev	Cell (excluding nucleus), nucleus, H+N, euchromatin	Standard deviation of intensity of the segmented object
intensity_median	Cell (excluding nucleus), nucleus, H+N, euchromatin	Median intensity of the segmented object
intensity_iqr	Cell (excluding nucleus), nucleus, H+N, euchromatin	Interquartile range (iqr) of intensity of the segmented object
intensity_total	Cell (excluding nucleus), nucleus, H+N, euchromatin	Sum of intensity values of the segmented object
Intensity_mean_25<br>Intensity_mean_50<br>Intensity_mean_75<br>Intensity_mean_100	Nucleus	Mean intensity of different radial zones of the nucleus.
Intensity_st_dev_25<br>Intensity_st_dev_50<br>Intensity_st_dev_75<br>Intensity_st_dev_100	Nucleus	Standard deviation of intensity of different radial zones of the nucleus.

Intensity_median_25<br>Intensity_median_50<br>Intensity_median_75<br>Intensity_median_100	Nucleus	Median intensity of different radial zones of the nucleus.
Intensity_iqr_25<br>Intensity_iqr_50<br>Intensity_iqr_75<br>Intensity_iqr_100	Nucleus	Interquartile range (iqr) of intensity of different radial zones of the nucleus.
Intensity_total_25<br>Intensity_total_50<br>Intensity_total_75<br>Intensity_total_100	Nucleus	Sum of intensity values of different radial zones of the nucleus.
Texture_hara1<br>Texture_hara2<br>Texture_hara3<br>Texture_hara4<br>Texture_hara5<br>Texture_hara6<br>Texture_hara7<br>Texture_hara8<br>Texture_hara9<br>Texture_hara10<br>Texture_hara11<br>Texture_hara12<br>Texture_hara13	Cell (excluding nucleus), nucleus, H+N, euchromatin	Haralick texture features of the segmented object. Haralick texture features 1-13 are commonly used texture descriptors in image analysis - each is a statistic derived from the grey level co-occurrence matrix of an image. (Haralick et al., 1973)
Shape_edt_mean_(het/eu)_nucleus	H+N, euchromatin	Mean of values in normalised euclidean distance transform (edt) of whole nucleus covered by the current segmented object (either euchromatin or heterochromatin + nucleolus segmentation). This is a measure of the distribution of chromatin within the nucleus (low values indicate a distribution mostly towards the edge of the nucleus, while higher values indicate a distribution closer to the centre). The euclidean distance transform is calculated for the whole nucleus segmentation, and normalised to run from 0 to 1 (1 being the point furthest from the edge).
Shape_edt_stdev_(het/eu)_nucleus	H+N, euchromatin	Standard deviation of values in normalised euclidean distance transform (edt) of whole nucleus covered by the current segmented object (either euchromatin or heterochromatin + nucleolus segmentation). This is a measure of the distribution of chromatin within the nucleus (high values indicate a varied distribution in the nucleus i.e. some towards the outside of the nucleus, as well as some towards the centre). The euclidean distance transform is calculated for the whole nucleus segmentation, and normalised to run from 0 to 1 (1 being the point furthest from the edge).
Shape_edt_median_(het/eu)_nucleus	H+N, euchromatin	Same as shape_edt_mean_(het/eu)_nucleus, but using median rather than the mean
Shape_edt_iqr_(het/eu)_nucleus	H+N, euchromatin	Same as shape_edt_stdev_(het/eu)_nucleus, but using interquartile range (iqr) rather than standard deviation.

Shape_percent_25_(het/eu)_nucleus<br>Shape_percent_50_(het/eu)_nucleus<br>Shape_percent_75_(het/eu)_nucleus<br>Shape_percent_100_(het/eu)_nucleus	H+N, euchromatin	Percent of different radial zones in the nucleus that are filled by either the heterochromatin+nucleolus or euchromatin segmentation - this is a measure of the distribution of chromatin within the nucleus.

Supplementary Table 1: Description of morphological features used in clustering analysis. Related to Figure 3.

Description of morphological, intensity and texture features used in clustering analysis. Note: some features are calculated for a number of different segmentations (as indicated by the second column), so e.g. Shape_volume_in_microns is calculated for 4 segmentations, and results in 4 features in the final table. Het / eu in feature names are short for 'heterochromatin + nucleolus' (H+N) and 'euchromatin' respectively. Some intensity and texture features are calculated for 'Cell (excluding nucleus)' i.e. the nucleus segmentation is used to mask out that part of the cell, and allow the measurement to be made only for the cytoplasm and organelles. Some features are measured within 'radial zones' of the nucleus - these zones are calculated from a euclidean distance transform of the nucleus, that is normalised to run from 0 to 1 (1 being the point in the nucleus furthest from the edge). Zone 25 is then the outermost 25% (values 0-0.25), zone 50 from 25% to 50% (values 0.25-0.5), zone 75 from 50% to 75% (values 0.5-0.75) and zone 100 from 75% to 100% (0.75-1.0).
The full table of calculated features for cells is available here: https://github.com/mobie/platybrowser-datasets/blob/master/data/1.0.1/tables/sbem-6dpf-1-whole-segmented-cells/morphology.csv and for nuclei (+ chromatin) here: https://github.com/platybrowser/platybrowser-backend/blob/master/data/1.0.0/tables/sbem-6dpf-1-whole-segmented-nuclei/morphology.csv. The clustering derived from both these sets of features combined is available here: https://github.com/mobie/platybrowser-datasets/blob/master/data/1.0.1/tables/sbem-6dpf-1-whole-segmented-cells/morphology_clusters.csv
